# From vibrations to function: Spectroscopic detection and quantification of π-π stacking in drug-responsive protein complexes

**DOI:** 10.1126/sciadv.aeb3917

**Published:** 2026-04-08

**Authors:** Narangerel Altangerel, Esther J. Ocola, Benjamin W. Neuman, Vladislav V. Yakovlev, Syuan-Ting Kuo, Veerabhadra Reddy Vulupala, Kaustav Khatua, Hanyuan Zhang, Xin Yan, David H. Russell, Shiqing Xu, Carol A. Fierke, Wenshe Ray Liu, Alexei V. Sokolov, Philip R. Hemmer, Marlan O. Scully

**Affiliations:** ^1^ Texas A&M University, College Station, TX 77843, USA.; ^2^ Brandeis University, Waltham, MA 02453, USA.; ^3^ Baylor University, Waco, TX 76798, USA.

## Abstract

Aromatic π-π stacking interactions are fundamental to protein architecture, molecular recognition, and drug efficacy, yet directly quantifying them under near-physiological conditions has remained challenging. Here, we use a recently developed spectroscopic platform, thermostable Raman interaction profiling (TRIP), that enables direct, label-free detection and quantification of aromatic π-π interactions in complex protein environments. Using the SARS-CoV-2 (severe acute respiratory syndrome coronavirus 2) main protease (M^pro^) as a biologically and clinically relevant model, we demonstrate that subtle changes in the phenylalanine benzene ring breathing (BRB) mode serve as a precise spectroscopic indicator of π-π stacking strength. This signal is highly responsive to both protein concentration-dependent dimerization and ligand-induced structural changes. M^pro^ forms a catalytically active dimer stabilized by a conserved aromatic triad (phenylalanine-140, histidine-163, and histidine-172), providing an ideal system to interrogate π-stacking at an important protein interface. Potent inhibitors MPI8 and nirmatrelvir produced the strongest BRB spectral shifts, broadening, and intensity changes, consistent with enhanced aromatic stacking and dimer stabilization, whereas halicin and VB-B-145 showed weaker engagement. BRB spectral changes also showed quantitative correlation with dimerization efficiency, published IC_50_ (median inhibitory concentration) values, and antiviral efficacy in A549-ACE2 cells. Complementary density functional theory revealed electron density rearrangements and vibrational coupling patterns unique to stacked aromatic residues. This integrated spectroscopic-computational approach enables quantitative probing of π-π stacking in native-like protein environments and positioning TRIP as a generalizable tool for designing drugs targeting aromatic protein-protein interfaces.

## Teaser

TRIP measures aromatic π-π protein interactions under near-physiological conditions, enabling structure-guided antiviral design.

## INTRODUCTION

Aromatic π-π interactions, also known as π-stacking, are noncovalent attractive forces between aromatic rings. These interactions arise from the London dispersion forces and dipole-induced dipole interactions between the delocalized π-orbital electrons in aromatic rings. Since the first recognition that those interactions might play a leading role in the shape and stability of life’s essential molecules, proteins, nucleic acids, and lipids (*1*), there has been a growing interest in understanding a rather controversial physical nature of such interactions (*2*, *3*) and its applications (*4*) due to their ability to facilitate molecular recognition and modulate binding affinity and selectivity in drug targets (*5*, *6*).

Despite their ubiquity in crystal structures, docking models, and mechanistic illustrations, the fundamental biophysical principles underlying π-π stacking in physiological aqueous environments remain poorly understood. Key questions persist: What determines stacking geometry preference in proteins? How strong are π-π interactions under native conditions? And to what extent do they influence structural stability, ligand affinity, or biological activity? These unanswered questions underscore a substantial gap in our comprehension of noncovalent interactions in biological systems.

**Fig. 1.**
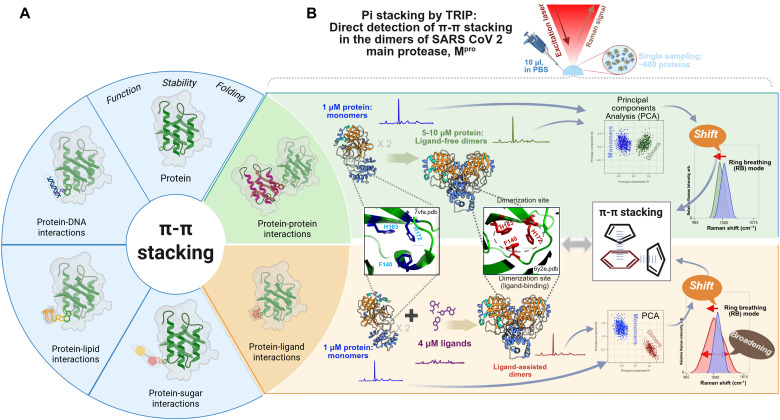
Direct profiling of aromatic π-π stacking in protein complexes using TRIP. (**A**) Aromatic π-π stacking interactions exist in protein and protein complexes. (**B**) Overview of the workflow using TRIP for direct measurement of π-π stacking in the dimers of SARS-CoV-2 main protease, M^pro^. Created in BioRender. Altangerel, N. (2026) https://BioRender.com/i5b5fx0. arb., arbitrary units.

Fundamentally, π-π interactions are notably complex. Unlike directional and relatively well-characterized covalent and hydrogen bonds (*7*), π-π stacking involves multiple geometries (parallel, T-shaped, and offset) and is strongly influenced by solvent conditions, such as ionic strength and molecular polarizability (*8*). On the practical side, π-π interactions are of the paramount significance for the rational design of inhibitors targeting protein-protein interfaces, especially when traditional binding sites are absent. These interactions play pivotal roles in assembling viral proteases, amyloid fibrils, and biomolecular condensates, positioning them as structural motifs of therapeutic interest (*9*–*11*). Figure 1A illustrates the scenarios relevant to protein or protein complexes where π-π stacking interactions can occur.

Now, most π-π interactions are inferred indirectly rather than measured directly with the exception of single-molecule force microscopy approaches (*12*), which, however, introduce substantial limitations on the type of molecules that can be studied. Various techniques have been used to probe π-π stacking, each with distinct advantages and limitations, and table S1 summarizes existing techniques. X-ray crystallography provides atomic-level insight into aromatic stacking geometries within static crystalline environments (*5*). Nuclear magnetic resonance (NMR) spectroscopy offers dynamic perspectives on π-π interactions in aqueous solutions but struggles to isolate specific stacking events clearly (*13*). Cryo–electron microscopy (cryo-EM) captures π-π interactions at atomic resolution but is limited to frozen, nondynamic states (*14*). Mass spectrometry indirectly assesses stacking through complex stability measurements yet lacks direct spatial resolution (*15*). Molecular dynamics (MD) simulations computationally explore π-π interactions dynamically, although they depend heavily on accurate parameterization and experimental validation (*16*). Fluorescence spectroscopy indirectly detects stacking interactions via fluorescent labeling, potentially altering native interactions (*17*). Similarly, ultraviolet (UV) spectroscopy relies on labeled spectral shifts to infer stacking, reducing direct interpretability (*18*). UV resonance Raman spectroscopy directly captures aromatic stacking via specific vibrational shifts but requires resonance conditions and may lack physiological compatibility (*19*). Surface-enhanced Raman spectroscopy (SERS) indirectly detects stacking through enhanced signals using nanoparticles, potentially perturbing native interactions (*20*). As a result, much of our current understanding of π-π interactions relies more on visual intuition than on quantitative experimental evidence.

To address these limitations, we use thermostable Raman interaction profiling (TRIP), which we have recently developed and validated for solution-phase, label-free spectroscopic measurements with unprecedented precision and accuracy (*21*). It is uniquely suited for direct detection and quantification of the strength of aromatic π-π interactions in proteins under near-physiological conditions. TRIP allows precise assessment of vibrational frequency shifts, such as the benzene ring breathing (BRB) mode of phenylalanine, which serve as a sensitive reporter of π-π stacking involving conserved aromatic residues.

We applied TRIP to investigate severe acute respiratory syndrome coronavirus 2 (SARS-CoV-2) main protease (M^pro^), a critical viral enzyme whose functional dimer is stabilized by a π-stacking trimer composed of Phe^140^, His^163^, and His^172^ (Fig. 1B). M^pro^ presents an excellent model system for probing π-π stacking interactions, owing to its well-defined dimerization interface and clinical significance (*22*). Among these residues, Phe^140^ and His^163^ are fully conserved across all known coronavirus M^pro^ sequences, suggesting a key role in dimerization-dependent activation of the enzyme (fig. S1). His^172^, although slightly less conserved, is still present in ~86% of M^pro^ sequences, including all representatives of the Orthocoronavirinae subfamily, which encompasses all known mammalian and avian coronaviruses such as SARS-CoV (severe acute respiratory syndrome coronavirus), SARS-CoV-2, and MERS-CoV (*23*, *24*). His^172^ is also broadly conserved in coronavirus clades beyond Orthocoronavirinae.

Functional mutagenesis studies have highlighted the critical nature of these aromatic residues. Mutation of His^163^ to alanine disrupted the π-stacking network by causing Phe^140^ to reorient away from the interface, destabilizing the active site and leading to significant conformational changes (*25*) and consistent with previous results for the equivalent histidine in other coronaviruses (*26*). Comprehensive mutagenesis and functional characterization of the SARS-CoV-2 protease enzyme has shown that Phe^140^ and His^172^ were relatively intolerant of amino acid substitutions and His^163^ was completely intolerant of any substitution (*24*). These findings underscore the essential role of aromatic π-π interactions—particularly those involving highly conserved residues—in maintaining the structural integrity and functional dimerization of M^pro^ across diverse coronavirus lineages.

Using TRIP, we detect ligand-induced spectral changes that report directly on π-π stacking strength at the dimer interface. These changes correlate linearly with M^pro^ dimerization, median inhibitory concentration (IC_50_) values, and antiviral efficacy. To interpret the molecular origins of these shifts, we integrate TRIP with density functional theory (DFT) calculations that model how stacking geometry influences electron density and vibrational coupling. This combined TRIP-DFT approach fills a long-standing gap by enabling quantitative assessment of π-π stacking strength in solution and establishing the Phe^140^ aromatic interface as a drug-targetable structural feature.

Beyond these structural implications, the functional significance of π-stacking has been largely unexplored. A central open question is whether the magnitude of stacking interactions can predict biochemical potency or antiviral activity. Because stacking-mediated stabilization is a defining feature of the M^pro^ dimer, we hypothesized that TRIP-derived BRB signatures could serve as functional predictors of inhibitor efficacy. This study therefore examines whether π-stacking strength at Phe^140^ correlates with enzymatic inhibition and cellular antiviral response, establishing a direct link between an experimentally measurable aromatic interaction and biological outcome.

Notably, DFT calculations of the F140L (Phe^140^ → Leu) aromatic-loss mutant demonstrate that removal of the Phe^140^ aromatic ring abolishes the BRB perturbation, reinforcing that this vibrational signature specifically reports π-π stacking at the dimer interface.

Last, because TRIP is inherently sensitive to π-stacking, we compare TRIP-based predictions with DFT models that capture the full interaction landscape. This dual experimental-computational strategy enables us to distinguish inhibitors whose potency derives from aromatic stacking.

## RESULTS

### Distinguishing M^pro^ monomers and reversible dimers via TRIP and DFT

#### Mass spectrometric determination of monomer and dimer concentrations

To determine the concentration thresholds that define the monomeric and dimeric states of the SARS-CoV-2 main protease M^pro^, we used variable-temperature electrospray ionization mass spectrometry (*27*) (vT-ESI-MS), a technique widely used for assessing protein oligomerization. At a concentration of 0.5 μM, M^pro^ was predominantly monomeric at 4°C, with ~50% of the monomers persisting even at room temperature (fig. S2A). In contrast, at 4 μM, more than half of the protein remained monomeric at 4°C, but the proportion of monomers dropped to below 20% at room temperature, indicating a significant shift toward dimerization (fig. S2A). The dissociation constant (*K*_d_) values (*28*) derived from these measurements were calculated to be 8.78 μM at 4°C and 0.47 μM at 25°C, underscoring the strong temperature dependence of the monomer-dimer equilibrium (fig. S2B).

To ensure experimental consistency, all subsequent TRIP experiments were performed at a stable temperature of 12°C. On the basis of the mass spectrometry data, we selected three M^pro^ concentrations in phosphate buffer solution (PBS) for TRIP analysis: 1 μM to represent predominantly monomeric conditions, 5 μM to represent a mixed monomer-dimer state, and 10 μM to reflect a largely dimeric population.

#### TRIP analysis of the M^pro^ monomer-dimer equilibrium

**Fig. 2.**
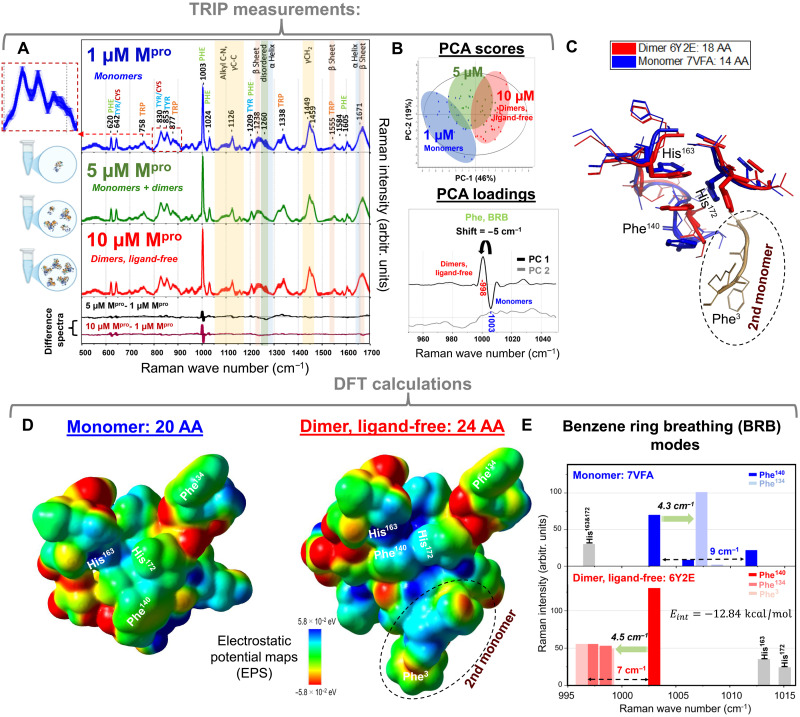
Spectroscopic and computational analysis of π-π stacking in SARS-CoV-2 M^pro^. (**A**) TRIP-measured Raman spectra of SARS-CoV-2, main protease M^pro^ in PBS with their SEs (shaded: *n*_each_ = 25 to 30; see the zoomed image): 1 μM concentrated (blue curves), 5 μM concentrated (green curves), and 10 μM concentrated (red curves), the difference spectrum between average Raman spectra of 5 and 1 μM (black curve), and the difference spectrum between average Raman spectra of 10 and 1 μM (maroon curve). (**B**) PCA score plots of the TRIP-measured Raman spectra of 1 μM (blue dots), 5 μM (green dots), and 10 μM (red dots) of SARS-CoV-2, main protease M^pro^ solutions, and their corresponding loadings. (**C**) Overlapped x-ray images of the M^pro^ monomer, 7vfa (blue) and the M^pro^ ligand-free dimer, 6y2e (red) (zoomed into the dimerization site of M^pro^). (**D**) Maps of ESP for the M^pro^ monomer (7vfa.pdb: Asn^133^-Gly^147^, His^162^-Met^164^, and Val^171^-Ala^173^) and the M^pro^ ligand-free dimer [6y2e.pbd: monomer 1 (Asn^133^-Gly^147^, His^162^-Met^164^, and Val^171^-Ala^173^) and monomer 2: Ser^1^-Arg^4^] calculated by DFT (Gaussian 16/B97D/DEF2SVP) in water (the closest solvent option available for PBS). The ESP maps plotted using the same color range from red (5.8 × 10^−2^ eV, negative) to blue (5.8 × 10^−2^ eV, positive). The red and blue colors in the ESP maps represent the lowest and highest ESP energy values, respectively. (**E**) DFT (Gaussian 16/B97D/DEF2SVP)–calculated Raman modes of the BRB for the monomer (Phe^140^: blue bars and Phe^134^: light blue bars; scaling factor: 1.025) in water and the dimer (Phe^140^: red bar, Phe^134^: medium red bar, and Phe^3^: light red bar; scaling factor: 1.015) in water. AA, amino acids; arbitr. units, arbitrary units.

TRIP was applied to assess whether it could distinguish between monomeric and dimeric M^pro^ forms by analyzing Raman spectra acquired at the selected concentrations of 1, 5, and 10 μM (Fig. 2A).

The Raman modes of protein samples are extensively studied and well known (*21*, *29*–*31*). The difference spectra revealed notable changes in the BRB mode of phenylalanine at ~1003 cm^−1^, which serves as a vibrational signature of π-π interactions (Fig. 2A). Principal components analysis (PCA) conducted on the spectral range of 950 to 1050 cm^−1^ showed that the spectra corresponding to 1 and 10 μM M^pro^ clustered distinctly, indicating clear spectral differences between monomer-dominant and dimer-dominant conditions in Fig. 2B. Considering the Raman data collected on three different sampling days, PCA clearly clustered replicates from the same concentration together, indicating high reproducibility of the spectral measurements. The 5 μM spectra overlapped both groups, consistent with an intermediate monomer-dimer distribution. Analysis of the first principal component revealed a downshift of about 5 cm^−1^ in the phenylalanine BRB mode, from 1003 to 998 cm^−1^, at higher protein concentrations. This shift is indicative of vibrational strain resulting from dimer formation. Furthermore, the increased intensity of the BRB peak in the dimeric samples suggests a modification in the local hydrophobic environment around phenylalanine residues (*32*).

#### DFT calculations of the monomer and ligand-free dimer

Aromatic π-π interactions are often dominated by dispersion forces, which pose a challenge for many traditional electronic structure methods. Fortunately, the past decade has seen substantial progress in developing efficient and accurate approaches for modeling such noncovalent interactions, as highlighted in several recent reviews (*33*, *34*). Among the most notable are DFT methods that incorporate dispersion effects. To support and interpret the TRIP observations, we performed DFT calculations using crystallographic structures of monomeric (7VFA.pdb) and dimeric (6Y2E.pdb) M^pro^. Structural alignment of the monomer and dimer (Fig. 2C) revealed that three key aromatic residues—Phe^140^, His^163^, and His^172^—form a π-π stacking trimeric interface in the dimer. Overlaying monomeric and dimeric x-ray structures (Fig. 2C) revealed distinct conformational differences in these residues, suggesting a previously unrecognized T-shaped π-π stacking interaction between Phe^140^ and His^172^, in addition to the known parallel π-π stacking between Phe^140^ and His^163^. Upon our review, this trimeric arrangement was consistently observed in other SARS-CoV-2 M^pro^ structures (see fig. S1). Also, a model generated by the AlphaFold 3 (without any constrains) had this trimer (see fig. S3).

In this work, we primarily rely on DFT calculations supplemented with Grimme’s empirical “D2” dispersion correction (*35*). Specifically, we use the B97-D functional, which combines Becke’s generalized gradient approximation (GGA) functional B97 (*36*) with the D2 correction. Although the triple zeta valence (TZV) basis set (*37*, *38*) is often used for accurate treatment of noncovalent interactions, we found it computationally prohibitive for systems exceeding 300 atoms. As a compromise between accuracy and efficiency, we adopted the smaller polarized split valence (def2-SVP) basis set (*39*), which enabled tractable calculations, whereas its combination with B97-D function still captured the essential features of dispersion-driven interactions.

We limited our DFT models to include 295 atoms for the monomer and 360 atoms for the dimer, covering key residues from the dimerization interface (Asn^133^-Gly^147^, His^162^-Met^164^, Val^171^-Ala^173^, and Ser^1^-Arg^4^ for the dimer). All calculations were performed using the B97-D functional with the def2-SVP basis set in an implicit aqueous environment (water) to reflect PBS conditions using the Gaussian 16 (*40*). The software does not offer a specific option for PBS solvation, so we selected water as the closest available alternative.

In these models, Phe^134^ served as uninteracted (control) phenylalanine residue, whereas Phe^140^ was involved in stacking interactions. The optimized structures and electrostatic maps revealed substantial charge redistribution around Phe^140^ in the dimer, with visible polarization driven by interactions with His^163^ and His^172^ (Fig. 2D). This contrasts with the monomer, in which the benzene ring of Phe^140^ remained unchanged.

Our vibrational frequency calculations showed that, in the monomer model, the BRB mode of Phe^134^ was upshifted by 4.3 cm^−1^ relative to Phe^140^ (Fig. 2E). In contrast, in the dimer model the BRB modes of Phe^134^ and Phe^3^ overlapped and were downshifted by 4.5 cm^−1^ from the Phe^140^ BRB mode, closely matching the ~5-cm^−1^ downshift observed experimentally in TRIP spectra. The BRB mode intensity also increased in the dimer, again consistent with TRIP measurements. For direct comparison, the calculated BRB frequency of Phe^140^ was scaled to 1003 cm^−1^ for all structures to align with the experimental position.

Because of its close proximity to the BRB mode, we also examined the imidazole ring deformation (IRD) mode. In the monomer, the IRD modes of His^163^ and His^172^ appeared 6 cm^−1^ below the Phe^140^ BRB mode, whereas in the dimer, they shifted to 10 to 12 cm^−1^ above the BRB mode. These opposing shifts indicate increased electronic coupling and the onset of π-π stacking interactions in the dimer.

The electrostatic potential (ESP) maps provide additional support for this interpretation. In the dimer (Fig. 2D), the ESP shows a pronounced pattern of alternating positive and negative regions distributed across Phe^140^, His^163^, and His^172^ when they are brought into close proximity. Specifically, the electron-rich π clouds of His^163^ and His^172^ align with the electron-deficient quadrupolar region of Phe^140^, forming a characteristic CH···π and quadrupole-quadrupole interaction network. This electrostatic signature is a well-established hallmark (*38*) of π-π stacking and is absent in the monomer ESP map, where the rings remain spatially separated (Fig. 2D).

Last, the calculated interaction (binding) energy of the dimer, obtained using eq. S1 (*41*) in the Supplementary Materials, was −12.84 kcal/mol. This strongly negative value further supports the presence of substantial aromatic interactions in the dimer.

#### Simplified DFT models: Aromatic dimers and trimer

To simplify and isolate the effects of π-π stacking interactions, we constructed phenylalanine-histidine dimer and trimer models and analyzed their vibrational and electrostatic properties. We began by examining noninteracting (free) phenylalanine and histidine molecules to establish a baseline. We then modeled two phenylalanine-histidine pairs separated by 5.8 and 7.2 Å, with atomic coordinates extracted from the monomeric M^pro^ structure (7VFA.pdb), to assess how these rings interact in the absence of π-π stacking. Subsequently, we constructed T-shaped and parallel-stacked dimer models using *XYZ* coordinates derived from the dimeric M^pro^ structure (6Y2E.pdb). These models allowed us to evaluate the individual contributions of each stacking geometry to electron density distribution and vibrational mode shifts. Last, we analyzed a trimeric aromatic stack—comprising Phe^140^, His^163^, and His^172^—to better understand the cooperative effects of multiring interactions observed in the dimeric M^pro^ conformation.

**Fig. 3.**
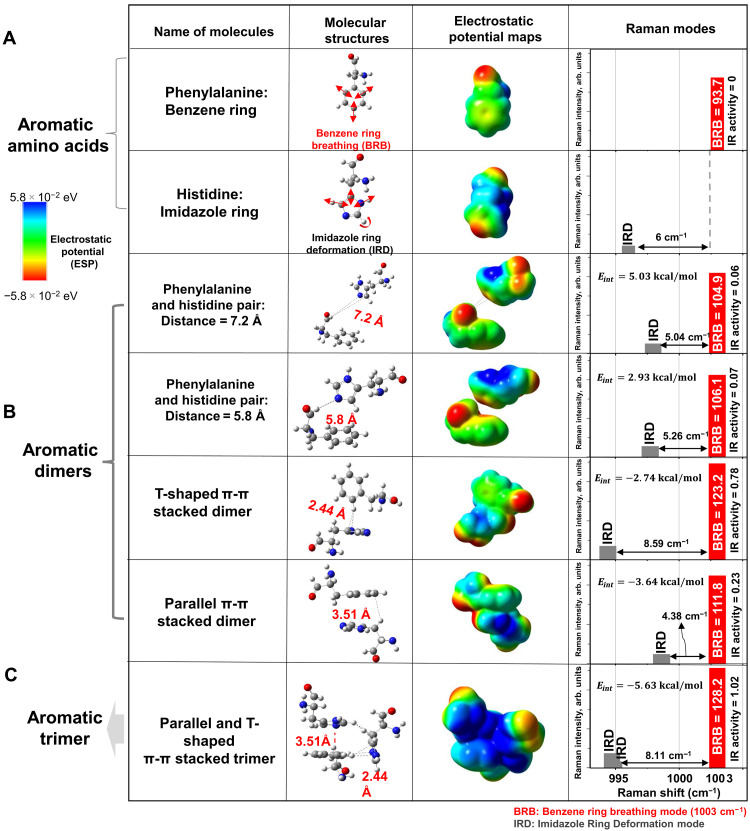
Structure-property relationships for aromatic interactions. (**A**) Free phenylalanine and histidine (scaling factors: 1.021 and 1.004). (**B**) Aromatic dimers: phenylalanine and histidine pair (distance = 7.2 Å; scaling factor: 1.02), phenylalanine and histidine pair (distance = 5.8 Å; scaling factor: 1.02), a T-shaped π-π stacked dimer (scaling factor: 1.017), and a parallel π-π stacked dimer (scaling factor: 1.022). (**C**) Aromatic trimer: phenylalanine and two histidine (scaling factor: 1.018); their *XYZ* coordinates are from the ligand free dimer M^pro^, 6y2e.pdb. ESP maps are plotted using the same color scheme (red: 5.8 × 10^−2^ eV, negative; blue: 5.8 × 10^−2^ eV, positive). The red and blue colors in the ESP maps represent the lowest and highest ESP energy values, respectively. Histograms of BRB and IRD modes are calculated by DFT (Gaussian 16/B97D/DEF2SVP) with water as the solvent. arb. units, arbitrary units.

We found that, in free phenylalanine and histidine molecules, the BRB and IRD modes were separated by ~6 cm^−1^ (Fig. 3A).

The vibrational modes of benzene ring, particularly its dominant BRB (*42*) mode, have been extensively calculated using DFT and experimentally validated both in isolated ring structures and within amino acid residues. Although the vibrational modes of imidazole rings have been studied, the IRD (*43*) mode—observed experimentally—has not been explicitly reported in previous DFT calculations due to its weak intensity. Consistent with this, our TRIP spectra of 180 mM phenylalanine, 180 mM histidine, and their mixture in PBS (fig. S4) showed that the IRD mode was approximately 30 times weaker than the BRB mode.

When the aromatic rings were brought closer (5.8 to 7.2 Å), the BRB-IRD mode separation decreased to 5.04 to 5.26 cm^−1^, although the ESP maps still showed clearly resolved electron density patterns (Fig. 3B). In this weakly interacting regime, the calculated interaction energies [using eq. S1 (*41*) in the Supplementary Materials] remained positive (2.93 and 5.03 kcal mol^−1^), consistent with the absence of π-π contact. In contrast, when the rings adopted well-defined π-π stacking geometries, the vibrational and energetic signatures changed markedly. In the T-shaped π-π stacked dimer, the IRD mode blue-shifted by 8.59 cm^−1^, whereas the parallel-stacked dimer exhibited a smaller 4.38-cm^−1^ blue shift. Correspondingly, the interaction energies of these dimers became negative (−2.74 and −3.64 kcal mol^−1^), indicating formation of stabilizing aromatic contacts. The aromatic trimer showed an intermediate but still substantial IRD blue shift of 8.11 cm^−1^ and the strongest binding energy [−5.63 kcal mol^−1^, calculated using eq. S2 (*41*)]. Together, these ESP and interaction energy patterns reinforce our earlier observations of the electrostatic signatures present in the monomer and ligand-free dimer models (Fig. 2D). The correlation between binding energy, BRB intensity, and IRD mode shift are shown in fig. S5.

The corresponding vibrational simulations reveal that π-π stacking not only induces blue shifts but also enhances the intensity of the BRB mode. In the T-shaped dimer, the −8.59-cm^−1^ BRB shift originates from electrostatic stiffening of the aromatic skeleton: The CH···π contact polarizes the imidazole π cloud and withdraws local electron density, increasing the C─N and C─C force constants. In contrast, the parallel dimer exhibits a smaller −4.38-cm^−1^ shift arising from partial inter-ring π-delocalization that slightly softens the C─C stretching network. The associated BRB intensity increases reflect the enhanced polarizability generated by π-orbital overlap.

In the benzene-imidazole-imidazole trimer, the parallel and T-shaped interactions act cooperatively, producing a highly anisotropic polarization of the benzene π cloud. This three-body effect withdraws π density from both imidazole rings, stiffening their C─N/C─C networks and generating the observed blue shifts in the IRD modes. The ESP map of the trimer (Fig. 3C) clearly visualizes the strong electron accumulation and polarization at the interfacial region, consistent with cooperative aromatic stabilization.

Among all models, the T-shaped configuration produces the strong polarization effect, yielding a threefold increase in infrared (IR) activity and a larger BRB-IRD separation than the parallel arrangement. The trimer exhibits even more extensive charge redistribution, with BRB IR activity rising to 1.02—an ~17-fold enhancement relative to the monomer model (0.06 to 0.07) (Fig. 3, B and C). Consistently, BRB intensity increases by 16% in the T-shaped dimer, 5% in the parallel dimer, and 21% in the trimer.

Together, these findings demonstrate that π-π stacking—particularly in the T-shaped geometry—substantially amplifies the vibrational response of phenylalanine and histidine, thereby improving their detectability via TRIP.

To confirm that these spectral shifts did not arise from histidine alone, we mentioned earlier that the imidazole ring’s IRD mode was much weaker than the BRB mode (fig. S4), confirming that the dominant spectral changes originate from π-π interactions rather than isolated histidine vibrations.

### Detecting ligand-assisted M^pro^ dimers via TRIP and DFT

**Fig. 4.**
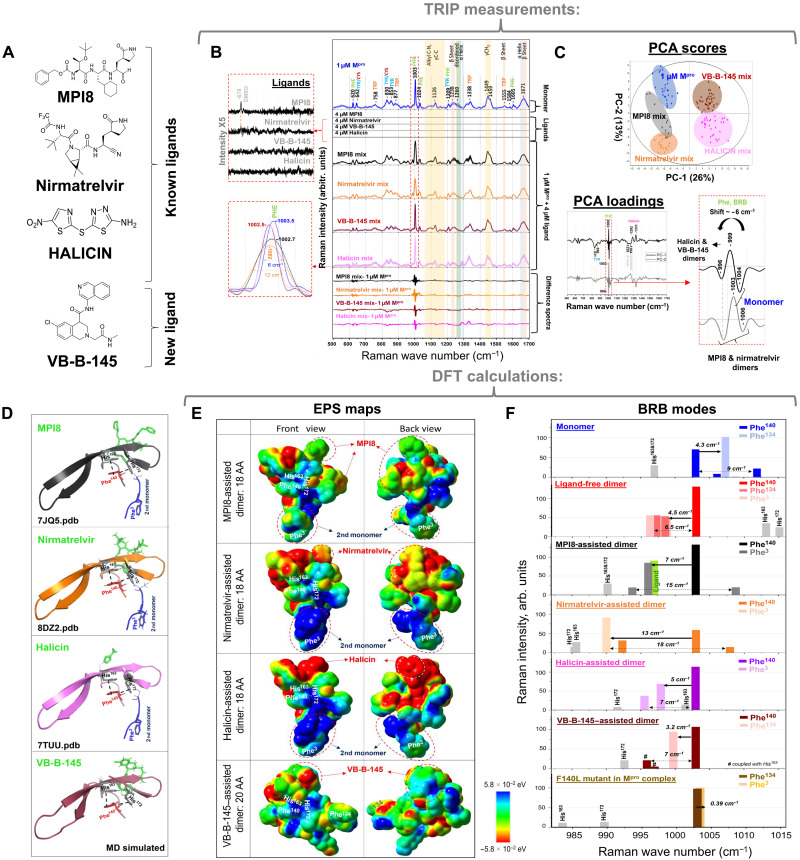
Structure and spectroscopy of inhibitors. (**A**) Chemical structures of inhibitors: known ligands of MPI8, nirmatrelvir, and halicin and a new ligand VB-B-145. (**B**) TRIP-measured Raman spectra with their SEs (shaded: *n*_each_ = 15 to 30): 1 μM M^pro^ solution (blue curves), 4 μM inhibitors (black curves), MPI8 mix (mix of 1 μM M^pro^ and 4 μM inhibitor) (black curves), nirmatrelvir mix (orange curves), halicin mix (pink curves), and VB-B-145 mix (maroon curves), and difference spectra between average Raman spectra of MPI8 mix and 1 μM (black curve), nirmatrelvir mix and 1 μM (orange curve), halicin mix and 1 μM (pink curve), and VB-B-145 mix and 1 μM (maroon curve). (**C**) PCA of the TRIP-measured Raman spectra of 1 μM (blue dots), MPI8 mix (black dots), nirmatrelvir mix (orange dots), halicin mix (pink dots), and VB-B-145 mix (maroon dots). (**D**) X-ray crystallography partial images of M^pro^ dimers (ligands are in light green): MPI8-assisted dimer in gray (PDB: 7JQ5), nirmatrelvir-assisted dimer in light orange (PDB: 8DZ2), halicin-assisted dimer in light violet (PDB:7TUU), and VB-B-145–assisted dimer in light maroon (MD simulated). (**E**) ESP maps for the dimer calculated by DFT (Gaussian 16/B97D/DEF2SVP). The red (−5.8 × 10^−2^ eV) and blue (+5.8 × 10^−2^ eV) colors in the ESP maps represent the lowest and highest ESP energy values, respectively. (**F**) BRB modes for the dimers calculated by DFT: MPI8-assisted dimer (Phe^140^: black and Phe^3^: gray bars; scaling factor: 1.013), nirmatrelvir-assisted dimer (Phe^140^: orange bars and Phe^3^: light orange bars; scaling factor: 1.007), halicin mix (Phe^140^: violet and Phe^3^: light violet bars; scaling factor: 1.015), VB-B-145–assisted dimer (Phe^140^: maroon and Phe^134^: light maroon bars; scaling factor: 1.017), and M^pro^ F140L mutant in complex, 8H4Y.pdb (Phe^134^: brown bars and Phe^3^: light brown bars; scaling factor: 1.021).

TRIP was then applied to investigate the binding of four inhibitors—MPI8, nirmatrelvir, halicin, and VB-B-145—to monomeric M^pro^. Whereas MPI8 (*44*), nirmatrelvir (*45*), and halicin (*46*) are known to form covalent bonds with Cys^145^, the binding characteristics of VB-B-145 were previously unknown. Notably, nirmatrelvir is the real antiviral component, an M^pro^ inhibitor, to the Food and Drug Administration (FDA)–approved antiviral combination therapy Paxlovid. The chemical synthesis of VB-B-145 is detailed in the Materials and Methods section (figs. S6 to S9), whereas the chemical structures of all inhibitors are shown in Fig. 4A.

MD simulations confirmed that VB-B-145 binds noncovalently via a water-mediated hydrogen bond to Cys^145^ (fig. S10).

#### TRIP measurements of bound structures

Raman spectra were collected under three conditions: M^pro^ alone at 1 μM, each inhibitor alone at 4 μM, and mixtures of M^pro^ with each inhibitor (1 μM + 4 μM) incubated for 24 hours at 4°C (Fig. 4B). The spectra of the inhibitors alone were largely featureless (Fig. 4B), confirming that spectral changes in the mixtures resulted from M^pro^-ligand interactions. Upon binding, all four inhibitors induced a downshift and reduction in intensity of the phenylalanine BRB mode, contrasting with the slight intensity increase seen in ligand-free dimers (Figs. 2A and 4B).

In particular, MPI8-bound and nirmatrelvir-bound M^pro^ showed significant broadening of the BRB peak, increasing from 6 to 12 cm^−1^, indicative of reduced protein backbone flexibility. PCA further distinguished monomeric and ligand-bound states as each complex formed separate clusters rather than overlapping with the monomer (Fig. 4C). All ligand-assisted dimers exhibited BRB downshifts of 4 to 6 cm^−1^, comparable to the shifts observed in natural dimerization (Figs. 2B and 4C).

PCA loadings revealed distinct spectral fingerprints for each ligand. MPI8 and nirmatrelvir displayed BRB peaks at 1006 and 996 cm^−1^, consistent with the broadening observed in their Raman spectra. These dimers also exhibited increased intensities of tyrosine Fermi resonance bands at 830 and 858 cm^−1^, which are known to be sensitive to hydrogen bonding. In the dimerization region, Tyr^126^ forms a hydrogen bond with Ser^139^ and Tyr^118^ with Lys^141^, which may contribute to these spectral changes (*47*).

Halicin induced a 4-cm^−1^ downshift in the BRB mode and introduced peaks at 1292 and 1331 cm^−1^, corresponding to its nitrothiazole ring deformation modes (Fig. 4B and fig. S11). VB-B-145 caused only a 4-cm^−1^ downshift in the BRB mode, confirming that it promotes M^pro^ dimerization despite lacking covalent binding.

#### DFT calculations of bound structures

DFT models of ligand-bound M^pro^ dimers were generated using crystallographic coordinates for MPI8 [Protein Data Bank (PDB): 7JQ5], nirmatrelvir (PDB: 8DZ2), and halicin (PDB: 7TUU), as well as MD-refined structures for VB-B-145. As illustrated in Fig. 4D (ligands are in light green; the partial structures: first monomer’s amino acids between 139 and 173 and second monomer’s amino acids between 1 and 5), all models consistently exhibit the formation of a conserved aromatic π-stacking trimer—Phe^140^, His^163^, and His^172^—at the dimerization interface. This π-trimer is structurally positioned on the inner surface of the second β barrel of the chymotrypsin-like domain. Although it does not make direct contact with the catalytic cysteine (Cys^145^), its proximity suggests a potential role in stabilizing the active site environment.

As previously noted, Phe^140^ and His^163^ are invariant across all known coronavirus M^pro^ sequences, whereas His^172^ is conserved in ~86% of cases. The active site of M^pro^ is formed by His^41^ and Cys^145^, with His^41^ extending from the first β barrel surface and Cys^145^ protruding from a flexible loop adjacent to the second β barrel, enabling their mutual orientation for catalytic activity (*47*). All three inhibitors—MPI8, nirmatrelvir, and halicin—form covalent bonds with Cys^145^, anchoring themselves away from the π-stacking region but within close spatial proximity, as depicted in Fig. 4D. Each DFT model explicitly includes Cys^145^ and the bound ligand, ensuring accurate representation of both catalytic and structural features of the dimer.

The VB-B-145–assisted dimer model included the ligand and 20 amino acids (Asn^133^-Gly^147^, His^162^-Met^164^, and Val^171^-Ala^173^), consistent with the monomer model described previously, due to the absence of a second monomer in the MD simulation. In contrast, the MPI8-assisted, nirmatrelvir-assisted, and halicin-assisted dimer models each included 18 amino acids (monomer 1: Ser^139^-Gly^147^, His^162^-Met^164^, and Val^171^-Ala^173^; monomer 2: Ser^1^-Arg^4^) along with their respective ligands.

Although the MPI8 model contained six fewer amino acids than the ligand-free dimer model, it had nearly the same total atom count (356 atoms versus 360), owing to the size of the MPI8 ligand (88 atoms). As shown in Fig. 2E, overlapping BRB modes of Phe^134^ and Phe^3^ in the ligand-free dimer confirmed that BRB mode of Phe^3^ can serve as a reference for phenylalanine residues not involved in stacking.

The DFT-predicted BRB shifts closely mirrored TRIP data: The MPI8-bound dimer showed a 7-cm^−1^ downshift, 13 cm^−1^ in nirmatrelvir, 5 cm^−1^ in halicin, and only 3.2 cm^−1^ in VB-B-145 (Fig. 4F). The relatively small shift observed in the VB-B-145 model is consistent with its noncovalent bond with Cys^145^ that mediated through a water molecule.

In addition, BRB mode bandwidths were notably wider for MPI8 (15 cm^−1^) and nirmatrelvir (18 cm^−1^), nearly double those of the monomer or other ligand-bound dimers, suggesting increased aromatic strain and reduced structural flexibility.

EPS maps derived from DFT calculations (Fig. 4E) revealed similar charge redistribution in the ligand-free dimer and the simplified models, particularly around the aromatic trimer formed by Phe^140^, His^163^, and His^172^. The maps show localized charge redistribution between the inhibitor’s aromatic moieties and the nearby phenylalanine residue, consistent with π-π stacking (Fig. 4E). The degree of polarization varies substantially across inhibitors: Halicin-assisted dimers exhibit the strongest ESP polarization, MPI8 and nirmatrelvir produce moderate polarization, whereas the VB-B-145–assisted dimer shows noticeably weaker polarization. Correspondingly, the BRB modes in the moderately polarized dimers (MPI8 and nirmatrelvir) display greater broadening and left-shifting, whereas the highly polarized (halicin) and weakly polarized (VB-B-145) dimers exhibit smaller shifts and reduced broadening. This nonmonotonic trend reflects the balance between electrostatic stiffening and vibrational mode delocalization as the aromatic environment becomes differently polarized.

Analysis of the IRD modes provided further mechanistic insight. In the monomer model, IRD modes were downshifted by 6 cm^−1^ relative to the BRB mode, similar to noninteracting histidine molecules. In ligand-free dimers, IRD modes were upshifted relative to BRB, indicating altered electronic coupling in the absence of bound ligands. Conversely, in all ligand-assisted dimers, the IRD modes were consistently downshifted relative to BRB. This shift pattern suggests that ligand-assisted dimers stabilize the aromatic interface more rigidly—likely corresponding to irreversible dimerization—whereas the upshift in ligand-free dimers may reflect their reversible, dynamic nature.

We mentioned earlier that the interaction energy of the ligand-free M^pro^ dimer was −12.84 kcal mol^−1^ using the standard two-body decomposition approach. This magnitude is consistent with typical noncovalent protein-protein interface stabilization energies. In contrast, attempting to compute binding energies for ligand-assisted dimers required geometry optimization of the protein interface in the absence of the ligand under identical computational conditions. Despite extensive attempts using the same B97-D/def2-SVP parameters that converged for the ligand-bound dimers, the ligand-free geometries of the MPI8-assisted, nirmatrelvir-assisted, and halicin-assisted models did not converge. This instability suggests that these ligand-assisted dimers are not structurally stable without the ligand present, consistent with their experimentally observed irreversible dimerization. Only the MD-refined VB-B-145 complex converged in the ligand-free state, yielding an apparent stabilization energy of −38.93 kcal mol^−1^—an unexpectedly large value for a noncovalent ligand interaction that likely reflects cooperative interface stabilization rather than a true ligand binding energy.

#### DFT calculation of the M^pro^ F140L mutant

To validate that the BRB frequency shift at residue 140 is a direct indicator of aromatic π-π interactions, we performed a DFT calculation using a model in which phenylalanine-140 was replaced with leucine (F140L). This DFT model was generated using crystallographic coordinates for the F140L mutant (PDB: 8H4Y.pdb) and included 21 amino acids (monomer 1: Phe^134^-Cys^145^, His^162^-Met^164^, and Val^171^-Ala^173^; monomer 2: Ser^1^-Phe^3^). As shown in Fig. 4F, the characteristic BRB shift observed in the native dimer was completely absent in this mutant. The BRB mode appeared unperturbed and closely matched the reference phenylalanine mode, confirming that the vibrational shift is strictly dependent on the presence of the aromatic π-π stacking interaction at residue 140. This result provides direct computational evidence that Phe^140^ is the essential driver of the π-stacking–induced BRB perturbation.

#### Multiple linear regression analysis of π-π stacking effects on M^pro^ composition and structure

To evaluate how π-π stacking influences the structural integrity of SARS-CoV-2 M^pro^, we applied our previously used the multiple linear regression (MLR) method to TRIP-derived Raman spectra (*48*). This method decomposes M^pro^ spectra into estimated amino acid compositions and secondary structure features using reference spectra from standard proteins and amino acids. Results showed that dimeric M^pro^ samples—especially those bound to VB-B-145 and halicin—exhibited modest reductions in residues like alanine, glycine, and threonine, indicating altered surface exposure or conformational dynamics comparable to ligand-free dimers (fig. S12A). In contrast, MPI8-bound and nirmatrelvir-bound dimers showed negligible changes in amino acids, reflecting strong stabilization of the conserved aromatic π-stacking trimer centered at Phe^140^ (fig. S12A). Key structural trends emerged from the MLR-derived secondary structure analysis of M^pro^ dimers (fig. S12B). First, all dimeric forms—whether ligand-free or ligand-assisted—exhibited a consistent decrease in β sheet content accompanied by an increase in α helix formation. This α helix/β sheet anticorrelation supports previous observations from circular dichroism (CD) and small-angle x-ray scattering (SAXS) studies (*49*), suggesting that dimerization drives M^pro^ toward a more compact, α helix–rich conformation. Second, a notable shift was observed in the balance between hydrogen-bonded turns and random coils. All ligand-bound dimers, with the exception of halicin, displayed a marked increase in H-bonded turns alongside a pronounced reduction in coil content (fig. S12B). This inverse trend—previously unreported for M^pro^—indicates that π-π stacking and ligand engagement promote local folding stabilization. The rise in hydrogen-bonded turns likely constrains loop flexibility, reducing disordered regions and enhancing overall structural order. These findings highlight how conserved aromatic interactions contribute not only to interface stabilization but also to broader secondary structure reorganization.

### Quantifying π-π stacking strength and its correlation with antiviral efficacy

#### Concentration-dependent Raman signatures of stacking disruption

**Fig. 5.**
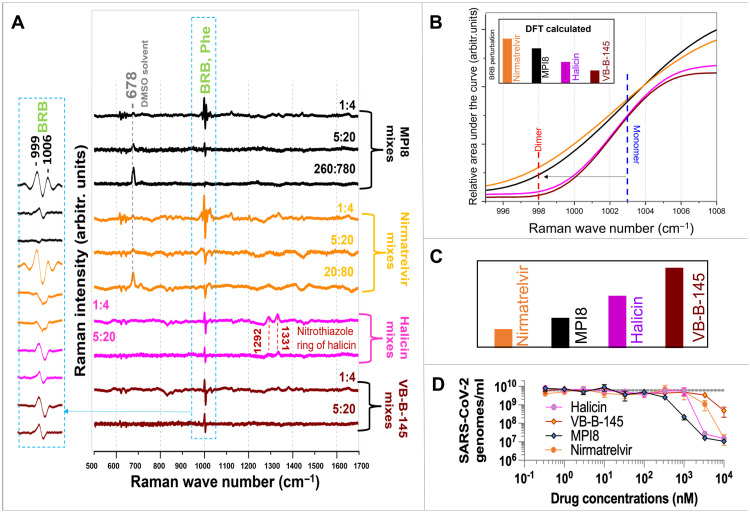
Assessment of the binding strength. (**A**) Difference Raman spectra between averages of Raman spectra of monomer M^pro^ and Raman spectra of the mixes: MPI8 mixes [1:4: 1 μM M^pro^ (mostly monomer) and 4 μM MPI8; 5:20: 5 μM M^pro^ (mix of a monomer and a dimer) and 20 μM MPI8; 260:780: 260 μM M^pro^ (dimers) and 780 μM MPI8) (black curves)], nirmatrelvir mixes (orange curves), halicin mixes (pink curves), and VB-B-145 mixes (maroon curves). (**B**) Absolute integrated area of the TRIP spectra within the phenylalanine BRB region for 1 μM ligand-assisted mixtures plotted as a function of wave number and BRB perturbations in the DFT calculations: MPI8 (black), nirmatrelvir (orange), halicin (light pink), and VB-B-145 (maroon). (**C**) IC_50_ values (*44*, *46*, *50*) shown as a bar graph: MPI8 (black), nirmatrelvir (orange), halicin (light pink), and VB-B-145 (maroon). (**D**) Enzyme inhibitions of M^pro^: M^pro^ activity is monitored in the presence of increasing concentrations of MPI8, nirmatrelvir, halicin, and VB-B-145.

To assess how ligands influence the π-π stacking interactions at the dimerization interface of SARS-CoV-2 M^pro^, we conducted a concentration-dependent TRIP analysis using four inhibitors: MPI8, nirmatrelvir, halicin, and VB-B-145. Ligand concentrations tested included 4 and 20 μM for all ligand-M^pro^ mixtures, with additional high-concentration conditions of 780 μM for MPI8 and 80 μM for nirmatrelvir. Difference Raman spectra (Fig. 5A) revealed subtle but informative changes as a function of ligand concentration, reflecting how π-π stacking interactions evolve with increasing ligand occupancy.

According to Fig. 5A, the most consistent and notable trend was a progressive reduction in the intensity and a slight downshift of the phenylalanine’s BRB mode at ~1003 cm^−1^. This vibrational mode is highly sensitive to π-π stacking interactions, especially those involving Phe^140^ at the M^pro^ dimer interface. The observed changes therefore directly reflect alterations in the electronic environment of the aromatic ring of Phe^140^ as dimerization is modulated by ligand binding. As the ligand concentration increased, the weakening of this π-π interaction—as evidenced by the BRB mode attenuation—suggested stabilization of the monomeric form of M^pro^.

At the highest concentrations of MPI8 and nirmatrelvir, an additional peak appeared at 678 cm^−1^, corresponding to the N─H stretching vibration of dimethyl sulfoxide (DMSO). This peak was more prominent in these samples due to their higher DMSO content from initial stock solutions (10 mM, 5× more diluted than halicin and VB-B-145 stock solutions). The absence of this peak in halicin and VB-B-145 mixtures at 20 μM confirmed that DMSO-associated spectral features were ligand-specific and concentration-dependent artifacts.

#### Ligand-dependent differences in monomer versus dimer preference

Comparative analysis of the concentration-dependent BRB mode changes across all ligand-M^pro^ mixtures revealed differential ligand binding preferences. Halicin displayed stronger interaction with monomeric M^pro^, as indicated by more prominent nitrothiazole ring deformation modes (1292 and 1331 cm^−1^) in monomer-rich samples (Fig. 5A). Our DFT calculations of halicin-assisted dimer model showed that these modes are present and much intensive than the BRB modes when the halicin bond with Cys^145^ (fig. S11) whereas unbound halicin’s TRIP spectra were almost flat (Fig. 4B).

VB-B-145 showed a stronger binding preference for the monomeric form, evidenced by a more substantial reduction in the BRB mode intensity when compared to dimer-containing mixtures. These patterns suggest that both halicin and VB-B-145 preferentially interact with monomeric M^pro^, whereas MPI8 and nirmatrelvir more effectively modulate the dimeric state by directly altering π-π stacking interactions at Phe^140^.

#### Absolute area under the BRB mode as π-π stacking metric and correlation with IC_50_

Next, we quantified the magnitude of π-π stacking by integrating the absolute area under the phenylalanine’s BRB vibrational region, a metric that captures both frequency shift and intensity change. The wave number–dependent area plots are shown in Fig. 5B. Larger BRB area values correspond to stronger π-π stacking at the dimer interface.

Among all inhibitors, MPI8 and nirmatrelvir produced the largest BRB shifts and intensity increases, indicating robust stabilization of the Phe^140^-centered π-π stacking network. Halicin showed low-intermediate stacking strength, whereas VB-B-145 exhibited the weakest aromatic engagement.

The reported IC_50_ values for nirmatrelvir, MPI8, and halicin are 66 nM (*50*), 105 nM (*44*), and 181.7 nM (*46*), respectively, and an unpublished value (fig. S13) for VB-B-145 is 279 nM. Because TRIP measurements require micromolar concentrations, we compared stacking strengths at 1 μM (Fig. 5B). At this accessible concentration, the BRB-derived stacking metric showed a clear monotonic relationship with inhibitory potency: Ligands that produced larger BRB area values exhibited lower IC_50_ values. Thus, TRIP-derived stacking strength predicts the potency ranking across all four inhibitors.

The BRB-based π-π stacking metric from TRIP correctly predicted the potency ranking for all four inhibitors: MPI8 > nirmatrelvir > halicin > VB-B-145. Ligands that induced larger BRB shifts and intensity changes consistently demonstrated stronger inhibitory activity.

To compare the DFT-derived vibrational signatures across different aromatic geometries, we calculated a BRB perturbation score defined as the product of the BRB frequency shift, the relative broadening of the band, and the maximum peak intensity. This empirically derived metric captures the combined spectral consequences of π-π stacking and allows direct comparison with experimentally observed BRB changes. In agreement with these experimental trends, the BRB perturbations predicted by our DFT models (Fig. 5B) produced the same potency order. Ligands that yielded larger calculated BRB shifts and greater mode coupling also exhibited lower IC_50_ values (Fig. 5C). The congruence between TRIP-derived measurements, DFT-calculated stacking perturbations, and IC_50_ values strengthens the conclusion that π-π stacking strength is a primary determinant of inhibitor efficacy at the M^pro^ dimer interface.

#### TRIP-derived stacking strength correlates with antiviral potency

To validate the biological significance of stacking strength, we compared TRIP-derived stacking metrics with antiviral efficacy data in A549-ACE2 cells infected with the SARS-CoV-2 Delta variant. Viral replication, assessed via RT-qPCR (reverse transcription quantitative polymerase chain reaction), revealed a strong correlation between π-π stacking strength and antiviral activity (Fig. 5D).

MPI8, which produced the strongest π-π stacking signatures, was also one of the most potent antivirals in the cellular assay. Nirmatrelvir exhibited slightly lower antiviral efficacy than MPI8, although its IC_50_ value is lower, indicating higher biochemical potency. This discrepancy likely reflects cell-dependent factors—such as permeability, metabolic stability, or efflux—that reduce intracellular drug concentrations independently of M^pro^ binding affinity. Halicin showed moderate antiviral activity consistent with its intermediate stacking strength, whereas VB-B-145 displayed the weakest stacking engagement and the lowest antiviral potency.

Minor discrepancies at higher ligand concentrations may arise from differences in cellular uptake or compound stability that are not directly detectable by TRIP. Additional factors outside of drug-M^pro^ affinity—including membrane permeability, metabolism, and efflux by transporters such as P-glycoprotein (P-gp), which is known to reduce intracellular concentrations of several drugs including nirmatrelvir (*51*)—can also influence antiviral efficacy.

By integrating TRIP with enzymatic and cellular assays, we demonstrate that the spectroscopically measured π-π stacking strength at Phe^140^ is a quantitative predictor of both biochemical inhibition (IC_50_) and antiviral efficacy in cells. MPI8 and nirmatrelvir, which induce the strongest stacking signatures, also exhibit the highest antiviral potency, whereas ligands with weaker stacking engagement show diminished cellular activity. These results establish aromatic stacking as not only a structural hallmark of the active M^pro^ dimer but also a functionally relevant interaction that directly influences therapeutic performance. This functional correlation elevates π-π stacking from a structural curiosity to a measurable biophysical determinant of drug efficacy, suggesting that TRIP-based stacking metrics could guide next-generation antiviral and interface-targeted drug design.

## DISCUSSION

Aromatic π-π interactions are inherently challenging to assess and quantify experimentally. In this study, we used state-of-the-art Raman spectroscopy, TRIP (*21*), complemented by DFT calculations, to provide insights into the nature and dynamics of these interactions. By integrating TRIP with DFT, we elucidated the quantum mechanical basis for stacking-induced charge redistribution and vibrational mode coupling. These calculations allowed to distinguish T-shaped and parallel π-π geometries and provided additional support that ligand binding induces electron density variations consistent with the observed changes of vibrational frequencies.

This work presents a comprehensive transformative investigation into the role of aromatic π-π interactions—specifically involving Phe^140^—in stabilizing the dimeric form of SARS-CoV-2 main protease M^pro^ and introduces TRIP as a label-free, solution-phase method for directly measuring these interactions. We show that ligand-induced changes in the phenylalanine BRB mode serve as a spectroscopic fingerprint of π-π stacking strength at the dimerization interface.

Using TRIP, we demonstrate that MPI8 and nirmatrelvir significantly enhance π-π stacking between Phe^140^, His^163^, and His^172^, as evidenced by BRB mode downshifts, intensity decreases, and spectral broadening. These changes correlate linearly with M^pro^ dimerization, IC_50_ values, and antiviral efficacy, establishing π-π stacking as a functionally predictive biophysical parameter. Halicin and VB-B-145, although capable of binding M^pro^, exhibit weaker π-π engagement and correspondingly reduced dimer stabilization.

MLR analysis of TRIP spectra further revealed that M^pro^ dimerization—particularly when stabilized by π-π stacking and ligand binding—induces a structural transition from β sheets to α helices, accompanied by an increase in hydrogen-bonded turns and a reduction in disordered coil regions.

Consistently, DFT-predicted BRB perturbations mirrored the experimentally measured IC_50_ values across all ligands, validating π-π stacking strength as a mechanistically relevant determinant of inhibitor potency. This alignment between theory, spectroscopy, and biological activity underscores the robustness of stacking-induced vibrational signatures as functional predictors.

Attempts to compute binding energies for ligand-assisted dimers provided additional mechanistic insight: Ligand-free geometries of the MPI8-associated, nirmatrelvir-associated, and halicin-associated dimers were unstable, whereas only the MD-refined VB-B-145 interface converged. This suggests that these dimers are intrinsically stabilized by their bound inhibitors.

DFT analysis of the F140L mutant further confirmed the aromatic origin of the BRB shift. Removal of the Phe^140^ aromatic ring abolished the π-stacking–induced vibrational perturbation, producing BRB frequencies indistinguishable from isolated phenylalanine. This demonstrates that the BRB shift is not a generic conformational effect but a specific spectroscopic hallmark of aromatic stacking at the dimer interface.

In summary, our findings provide a mechanistic and structural framework that connects aromatic π-π stacking with protein dimerization, ligand binding, conformational change, and antiviral activity. TRIP, when combined with quantum mechanical modeling and statistical deconvolution, unlocks π-π interactions as a tractable, quantifiable class of molecular forces in structural biology. This work lays the foundation for next-generation drug design strategies that target these subtle but powerful interactions to modulate protein-protein and protein-ligand assembly.

## MATERIALS AND METHODS

### Sample preparation

The expression and purification of SARS-CoV-2 M^pro^ were conducted according to the procedure in one previous report (*52*). MPI8 was synthesized according to one previous report (*52*). For the synthesis of VB-B-145, please refer to the Supplementary Materials. Halicin and nirmatrelvir were purchased without further purification. The original concentration of M^pro^ was 8.9 mg/ml, and it was diluted to 1, 5, and 10 μM in PBS for this study. The halicin and VB-B-145 solutions were 50 mM in DMSO. The nirmatrelvir and MPI8 solutions were supplied by 10 mM in DMSO. These solutions were further diluted to 20 and 4 μM in PBS. l-Phenylalanine (catalog no. P2126) and l-histidine (catalog no. H8000) were purchased from Sigma-Aldrich. The stock solutions of phenylalanine (302.7 mM, prepared in 1 M ammonium hydroxide) and histidine (322.3 mM, prepared in 1 M hydrochloric acid) were diluted to 180 mM using distilled water.

### Determination of *K*_d_ at variable temperatures by native mass spectrometry

M^pro^ was buffer exchanged to 200 mM ammonium acetate (pH 6.8) by using a Micro Biospin P-6 gel column (Bio-Rad) for mass spectrometry analysis. Native mass spectrometry (nMS) analysis was performed on a Q Exactive UHMR Hybrid Quadruple-Orbitrap Mass Spectrometer (Thermo Fisher Scientific) with the mass/charge ratio (*m/z*) range set from 1000 to 10,000. A 10-μl sample was loaded to a borosilicate glass capillary tip (Sutter, CA) with 1100- to 1500-V spray voltage supplied by an inserted platinum wire. Activation energies were carefully optimized to remove nonspecific adducts with minimal gas-phase activation. Those parameters include a capillary temperature of 100°C, in-source trapping and activation of −10 V, ion transfer set to high *m/z*, collision-induced dissociation (CID) of 10 eV, and higher-energy collisional dissociation (HCD) of 30 V. In the vT-ESI experiment (*27*, *28*), the temperature of the solution was controlled at 4° or 25°C and the time for equilibrium at each temperature was 5 min. The relative abundance of monomeric M^pro^ and dimeric M^pro^ were determined by deconvoluting the mass spectra with UniDec (*53*). The relative abundance was converted into concentration and subsequently used to yield the *K*_d_ as described in previous studies (*53*, *54*).

### Measurement protocol of the inhibition of M^pro^ activity by VB-B-145

A 20 nM solution of M^pro^ was incubated with VB-B-145 for 30 min before 10 μM Sub3 (DABCYL-Lys-Thr- Ser-Ala-Val-Leu-Gln-Ser-Gly-Phe-Arg-Lys-Met-Glu-EDANS) dissolved in DMSO. The production of fluorescent intensity with excitation at 336 nm and emission at 490 nm was measured 5 min after mixing, as described in (*55*).

### Apparatus and software

A confocal Raman microscope system (LabRAM: Horiba Inc.) was used for all spectroscopic studies. The excitation laser wavelength was 785 nm. For all protein samples, Raman spectral acquisitions were taken from a 10-μl drop of protein solution deposited on a gold-coated glass slide (Ted Pella no. 26002-G). A thin layer of gold on a glass slide served three purposes: (i) to dissipate thermal energy due to the optical absorption of the excitation laser, (ii) to block the fluorescent and Raman background signals from the glass substrate, and (iii) to reflect the forward scattered Raman signal toward the detector. The laser was focused to an ~1-μm spot size (full width at half maximum) inside the liquid samples using a 100 X microscope objective lens with 0.75 NA (numerical aperture). That means each sampling volume approximately contained about 660 M^pro^ proteins for the 1 μM M^pro^ concentration. The acquisition time was 5 s, and the signal was averaged over 12 spectra. The laser power at the sample was 7 mW. The detailed experimental setup is described in greater details in our previous report (*21*). The cooled stage was kept at a 12°C temperature on the gold substrate for all experimental samples.

The LabSpec 6 software was used to control the microscope, collect Raman spectra, and preprocessed the spectra. Data preprocessing included seventh-order polynomial background removal with 140 points, unit vector normalization, and Savitsky-Golay smoothing with 20 adjacent points. The Aspen Unscrambler software was used for PCA of Raman spectra of the experimental samples.

### DFT calculations

The calculation of different geometric configurations of the geometric structure and vibrational frequencies of several dimers of peptides has been calculated using the Gaussian 16/B97D/TZVP and B97D/DEF2SVP basis sets. The calculations were performed using the Gaussian16, Rev. C01 program (*40*), and the structures were visualized with the aid of GaussView 9.11 (*56*).

### The AlphaFold 3 model

We used the AlphaFold server (alphafoldserver.com) to model the ligand-free dimer by submitting monomer M^pro^ with its amino acid sequence of SGFRKMAFPSGKVEGCMVQVTCGTTTLNGLWLDDVVYCPRHVICTSEDMLNPNYEDLLIRKSNHNFLVQAGNVQLRVIGHSMQNCVLKLKVDTANPKTPKYKFVRIQPGQTFSVLACYNGSPSGVYQCAMRPNFTIKGSFLNGSCGSVGFNIDYDCVSFCYMHHMELPTGVHAGTDLEGNFYGPFVDRQTAQAAGTDTTITVNVLAWLYAAVINGDRWFLNRFTTTLNDFNLVAMKYNYEPLTQDHVDILGPLSAQTGIAVLDMCASLKELLQNGMNGRTILGSALLEDEFTPFDVVRQCSGVTFQ.

## References

[1] S. K. Burley, G. A. Petsko, Aromatic-aromatic interaction: A mechanism of protein structure stabilization. Science 229, 23–28 (1985). 3892686 10.1126/science.3892686

[2] C. R. Martinez, B. L. Iverson, Rethinking the term “pi-stacking”. Chem. Sci. 3, 2191–2201 (2012).

[3] C. D. Sherrill, Energy component analysis of π interactions. Acc. Chem. Res. 46, 1020–1028 (2013). 23020662 10.1021/ar3001124

[4] R. Thakuria, N. K. Nath, B. K. Saha, The nature and applications of π–π interactions: A perspective. Cryst. Growth Des. 19, 523–528 (2019).

[5] G. B. McGaughey, M. Gagné, A. K. Rappé, π-stacking interactions: Alive and well in proteins. J. Biol. Chem. 273, 15458–15463 (1998). 9624131 10.1074/jbc.273.25.15458

[6] C. A. Hunter, J. K. M. Sanders, The nature of π-π interactions. J. Am. Chem. Soc. 112, 5525–5534 (1990).

[7] D. Herschlag, M. M. Pinney, Hydrogen bonds: Simple after all? Biochemistry 57, 3338–3352 (2018). 29678112 10.1021/acs.biochem.8b00217

[8] E. A. Meyer, R. K. Castellano, F. Diederich, Interactions with aromatic rings in chemical and biological recognition. Angew. Chem. Int. Ed. Engl. 42, 1210–1250 (2003). 12645054 10.1002/anie.200390319

[9] A. Patel, L. Malinovska, S. Saha, J. Wang, S. Alberti, Y. Krishnan, A. A. Hyman, ATP as a biological hydrotrope. Science 356, 753–756 (2017). 28522535 10.1126/science.aaf6846

[10] E. Gazit, A possible role for π-stacking in the self-assembly of amyloid fibrils. FASEB J. 16, 77–83 (2002). 11772939 10.1096/fj.01-0442hyp

[11] D. Gonzalez-Ruiz, H. Gohlke, Targeting protein-protein interactions with small molecules: Challenges and perspectives for omputational binding epitope detection and ligand finding. Curr. Med. Chem. 13, 2607–2625 (2006). 17017914 10.2174/092986706778201530

[12] Y. Zhang, C. Liu, W. Shi, Z. Wang, L. Dai, X. Zhang, Direct measurements of the interaction between pyrene and graphite in aqueous media by single molecule force spectroscopy: Understanding the π−π interactions. Langmuir 23, 7911–7915 (2007). 17590031 10.1021/la700876d

[13] G. Platzer, M. Mayer, A. Beier, S. Brüschweiler, J. E. Fuchs, H. Engelhardt, L. Geist, G. Bader, J. Schörghuber, R. Lichtenecker, B. Wolkerstorfer, D. Kessler, D. B. McConnell, R. Konrat, PI by NMR: Probing CH–π interactions in protein–ligand complexes by NMR spectroscopy. Angew. Chem. Int. Ed. Engl. 59, 14861–14868 (2020). 32421895 10.1002/anie.202003732 PMC7496880

[14] Y. Cheng, Single-particle cryo-EM-how did it get here and where will it go. Science 361, 876–880 (2018). 30166484 10.1126/science.aat4346 PMC6460916

[15] S. Lenz, L. R. Sinn, F. J. O’Reilly, L. Fischer, F. Wegner, J. Rappsilber, Reliable identification of protein-protein interactions by crosslinking mass spectrometry. Nat. Commun. 12, 3564 (2021). 34117231 10.1038/s41467-021-23666-z PMC8196013

[16] M. Karplus, J. A. McCammon, Molecular dynamics simulations of biomolecules. Nat. Struct. Mol. Biol. 9, 646–652 (2002). 10.1038/nsb0902-646 12198485

[17] F. H. dos Santos Rodrigues, G. G. Delgado, T. Santana da Costa, L. Tasic, Applications of fluorescence spectroscopy in protein conformational changes and intermolecular contacts. BBA Adv. 3, 100091 (2023). 37207090 10.1016/j.bbadva.2023.100091 PMC10189374

[18] S. V. Thakkar, K. M. Allegre, S. B. Joshi, D. B. Volkin, C. R. Middaugh, An application of ultraviolet spectroscopy to study interactions in proteins solutions at high concentrations. J. Pharm. Sci. 101, 3051–3061 (2012). 22581726 10.1002/jps.23188

[19] S. A. Asher, UV resonance Raman spectroscopy for analytical, physical, and biophysical chemistry. Part 2. Anal. Chem. 65, 201A–210A (1993). 10.1021/ac00052a001 8439006

[20] M. Moskovits, Surface-enhanced spectroscopy. Rev. Mod. Phys. 57, 783–826 (1985).

[21] N. Altangerel, B. W. Neuman, P. R. Hemmer, V. V. Yakovlev, N. Rajil, Z. Yi, A. V. Sokolov, M. O. Scully, Label-free drug interaction screening via Raman microscopy. Proc. Natl. Acad. Sci. U.S.A. 120, e2218826120 (2023). 37463207 10.1073/pnas.2218826120 PMC10372630

[22] D. W. Kneller, S. Galanie, G. Phillips, H. M. O’Neill, L. Coates, A. Kovalevsky, Malleability of the SARS-CoV-2 3CL M^pro^ active-site cavity facilitates binding of clinical antivirals. Structure 28, 1313–1320.e3 (2020). 33152262 10.1016/j.str.2020.10.007 PMC7584437

[23] P. Garcia-Segura, A. Llop-Peiró, N. Novau-Ferré, J. Mestres-Truyol, B. Saldivar-Espinoza, G. Pujadas, S. Garcia-Vallvé, SARS-CoV-2 main protease (M-pro) mutational profiling: An insight into mutation coldspots. Comput. Biol. Med. 184, 109344 (2025). 39531923 10.1016/j.compbiomed.2024.109344

[24] J. M. Flynn, N. Samant, G. Schneider-Nachum, D. T. Barkan, N. K. Yilmaz, C. A. Schiffer, S. A. Moquin, D. Dovala, D. N. Bolon, Comprehensive fitness landscape of SARS-CoV-2 M^pro^ reveals insights into viral resistance mechanisms. eLife 11, e77433 (2022). 35723575 10.7554/eLife.77433 PMC9323007

[25] N. Tran, S. Dasari, S. A. E. Barwell, M. J. McLeod, S. Kalyaanamoorthy, T. Holyoak, A. Ganesan, The H163A mutation unravels an oxidized conformation of the SARS-CoV-2 main protease. Nat. Commun. 14, 5625 (2023). 37699927 10.1038/s41467-023-40023-4 PMC10497556

[26] J. Ziebuhr, G. Heusipp, S. G. Siddell, Biosynthesis, purification, and characterization of the human coronavirus 229E 3C-like proteinase. J. Virol. 71, 3992–3997 (1997). 9094676 10.1128/jvi.71.5.3992-3997.1997 PMC191551

[27] J. W. McCabe, M. Shirzadeh, T. E. Walker, C.-W. Lin, B. J. Jones, V. H. Wysocki, D. P. Barondeau, D. E. Clemmer, A. Laganowsky, D. H. Russell, Variable-temperature electrospray ionization for temperature-dependent folding/refolding reactions of proteins and ligand binding. Anal. Chem. 93, 6924–6931 (2021). 33904705 10.1021/acs.analchem.1c00870 PMC8119373

[28] T. Walker, H. M. Sun, T. Gunnels, V. Wysocki, A. Laganowsky, H. Rye, D. Russell, Dissecting the thermodynamics of ATP binding to GroEL one nucleotide at a time. ACS Cent. Sci. 9, 466–475 (2023). 36968544 10.1021/acscentsci.2c01065 PMC10037461

[29] T. Kitagawa, S. Hirota, “Raman spectroscopy of proteins” in *Handbook of Vibrational Spectroscopy* (John Wiley & Sons Ltd., 2006).

[30] T. Miura, G. J. Thomas, “Raman spectroscopy of proteins and their assemblies” in *Proteins: Structure, Function, and Engineering*, B. B. Biswas, S. Roy, Eds. (Springer US, 1995, pp. 55–99. 10.1007/978-1-4899-1727-0_3 7900183

[31] R. W. Williams, A. K. Dunker, Determination of the secondary structure of proteins from the amide I band of the laser Raman spectrum. J. Mol. Biol. 152, 783–813 (1981). 7334522 10.1016/0022-2836(81)90127-3

[32] Z.-Q. Wen, Raman spectroscopy of protein pharmaceuticals. J. Pharm. Sci. 96, 2861–2878 (2007). 17847076 10.1002/jps.20895

[33] K. E. Riley, M. Pitoňák, P. Jurečka, P. Hobza, Stabilization and structure calculations for noncovalent interactions in extended molecular systems based on wave function and density functional theories. Chem. Rev. 110, 5023–5063 (2010). 20486691 10.1021/cr1000173

[34] E. G. Hohenstein, C. D. Sherrill, Wavefunction methods for noncovalent interactions. Wiley Interdiscip. Rev. Comput. Mol. Sci. 2, 304–326 (2012).

[35] S. Grimme, Semiempirical GGA-type density functional constructed with a long-range dispersion correction. J. Comput. Chem. 27, 1787–1799 (2006). 16955487 10.1002/jcc.20495

[36] A. D. Becke, Density-functional thermochemistry. V. Systematic optimization of exchange-correlation functionals. J. Chem. Phys. 107, 8554–8560 (1997).

[37] A. Schäfer, C. Huber, R. Ahlrichs, Fully optimized contracted Gaussian basis sets of triple zeta valence quality for atoms Li to Kr. J. Chem. Phys. 100, 5829–5835 (1994).

[38] S. E. Wheeler, J. W. G. Bloom, Toward a more complete understanding of noncovalent interactions involving aromatic rings. J. Phys. Chem. A 118, 6133–6147 (2014). 24937084 10.1021/jp504415p

[39] F. Weigend, R. Ahlrichs, Balanced basis sets of split valence, triple zeta valence and quadruple zeta valence quality for H to Rn: Design and assessment of accuracy. Phys. Chem. Chem. Phys. 7, 3297–3305 (2005). 16240044 10.1039/b508541a

[40] M. Frisch, G. Trucks, H. B. Schlegel, G. E. Scuseria, M. Robb, J. R. Cheeseman, G. Scalmani, V. Barone, G. A. Petersson, H. Nakatsuji, X. Li, M. Caricato, A. Marenich, J. Bloino, B. Janesko, R. Gomperts, B. Mennucci, H. Hratchian, J. V. Ortiz, A. Izmaylov, J. Sonnenberg, D. Williams-Young, F. Ding, F. Lipparini, F. Egidi, J. Goings, B. Peng, A. Petrone, T. Henderson, D. Ranasinghe, V. G. Zakrzewski, J. Gao, N. Rega, G. Zheng, W. Liang, M. Hada, M. Ehara, K. Toyota, R. Fukuda, J. Hasegawa, M. Ishida, T. Nakajima, Y. Honda, O. Kitao, H. Nakai, T. Vreven, K. Throssell, J. A. Montgomery Jr., J. E. Peralta, F. Ogliaro, M. Bearpark, J. J. Heyd, E. Brothers, K. Kudin, V. Staroverov, T. Keith, R. Kobayashi, J. Normand, K. Raghavachari, A. P. Rendell, J. C Burant, S. Iyengar, J. Tomasi, M. Cossi, J. M. Millam, M. Klene, C. Adamo, R. Cammi, J. W. Ochterski, R. A. Martin, K. Morokuma, O. Farkas, J. Foresman, D. Fox, Gaussian 16 Revision C.01 (Gaussian Inc., 2016).

[41] A. J. Stone, *The Theory of Intermolecular Forces* (Oxford Univ. Press, 1996).

[42] A. Sakamoto, M. Tasumi, Symmetry of the benzene ring and its normal vibrations: The “breathing” mode is not always a normal vibration of a benzene ring. J. Raman Spectrosc. 52, 2282–2291 (2021).

[43] H. Takeuchi, Raman structural markers of tryptophan and histidine side chains in proteins. Biopolymers 72, 305–317 (2003). 12949821 10.1002/bip.10440

[44] X. R. Ma, Y. R. Alugubelli, Y. Ma, E. C. Vatansever, D. A. Scott, Y. Qiao, G. Yu, S. Xu, W. R. Liu, MPI8 is potent against SARS-CoV-2 by inhibiting dually and selectively the SARS-CoV-2 main protease and the host cathepsin L. ChemMedChem 17, e202100456 (2022). 34242492 10.1002/cmdc.202100456 PMC8427127

[45] S. Iketani, H. Mohri, B. Culbertson, S. J. Hong, Y. Duan, M. I. Luck, M. K. Annavajhala, Y. Guo, Z. Sheng, A.-C. Uhlemann, S. P. Goff, Y. Sabo, H. Yang, A. Chavez, D. D. Ho, Multiple pathways for SARS-CoV-2 resistance to nirmatrelvir. Nature 613, 558–564 (2023). 36351451 10.1038/s41586-022-05514-2 PMC9849135

[46] K. S. Yang, S.-T. Alex Kuo, L. R. Blankenship, Z. Z. Geng, S. G. Li, D. H. Russell, X. Yan, S. Xu, W. R. Liu, Repurposing Halicin as a potent covalent inhibitor for the SARS-CoV-2 main protease. Curr. Res. Chem. Biol. 2, 100025 (2022). 35815070 10.1016/j.crchbi.2022.100025 PMC9023366

[47] Z. Jin, X. Du, Y. Xu, Y. Deng, M. Liu, Y. Zhao, B. Zhang, X. Li, L. Zhang, C. Peng, Y. Duan, J. Yu, L. Wang, K. Yang, F. Liu, R. Jiang, X. Yang, T. You, X. Liu, X. Yang, F. Bai, H. Liu, X. Liu, L. W. Guddat, W. Xu, G. Xiao, C. Qin, Z. Shi, H. Jiang, Z. Rao, H. Yang, Structure of M^pro^ from SARS-CoV-2 and discovery of its inhibitors. Nature 582, 289–293 (2020). 32272481 10.1038/s41586-020-2223-y

[48] N. Altangerel, B. W. Neuman, P. R. Hemmer, V. V. Yakovlev, A. V. Sokolov, M. O. Scully, A novel non-destructive rapid tool for estimating amino acid composition and secondary structures of proteins in solution. Small Methods 8, e2301191 (2024). 38485686 10.1002/smtd.202301191 PMC11260246

[49] L. Silvestrini, N. Belhaj, L. Comez, Y. Gerelli, A. Lauria, V. Libera, P. Mariani, P. Marzullo, M. G. Ortore, A. Palumbo Piccionello, C. Petrillo, L. Savini, A. Paciaroni, F. Spinozzi, The dimer-monomer equilibrium of SARS-CoV-2 main protease is affected by small molecule inhibitors. Sci. Rep. 11, 9283 (2021). 33927258 10.1038/s41598-021-88630-9 PMC8085067

[50] Y. R. Alugubelli, Z. Z. Geng, K. S. Yang, N. Shaabani, K. Khatua, X. R. Ma, E. C. Vatansever, C.-C. Cho, Y. Ma, J. Xiao, L. R. Blankenship, G. Yu, B. Sankaran, P. Li, R. Allen, H. Ji, S. Xu, W. R. Liu, A systematic exploration of boceprevir-based main protease inhibitors as SARS-CoV-2 antivirals. Eur. J. Med. Chem. 240, 114596 (2022). 35839690 10.1016/j.ejmech.2022.114596 PMC9264725

[51] Y. Dönmez Cakil, N. Khunweeraphong, Z. Parveen, D. Schmid, M. Artaker, G. F. Ecker, H. H. Sitte, O. Pusch, T. Stockner, P. Chiba, Pore-exposed tyrosine residues of P-glycoprotein are important hydrogen-bonding partners for drugs. Mol. Pharmacol. 85, 420–428 (2014). 24366667 10.1124/mol.113.088526 PMC4503343

[52] K. S. Yang, X. R. Ma, Y. Ma, Y. R. Alugubelli, D. A. Scott, E. C. Vatansever, A. K. Drelich, B. Sankaran, Z. Z. Geng, L. R. Blankenship, H. E. Ward, Y. J. Sheng, J. C. Hsu, K. C. Kratch, B. Zhao, H. S. Hayatshahi, J. Liu, P. Li, C. A. Fierke, C.-T. K. Tseng, S. Xu, W. R. Liu, A quick route to multiple highly potent SARS-CoV-2 main protease inhibitors. ChemMedChem 16, 942–948 (2021). 33283984 10.1002/cmdc.202000924 PMC7979488

[53] L. A. Bergdoll, M. T. Lerch, J. W. Patrick, K. Belardo, C. Altenbach, P. Bisignano, A. Laganowsky, M. Grabe, W. L. Hubbell, J. Abramson, Protonation state of glutamate 73 regulates the formation of a specific dimeric association of mVDAC1. Proc. Natl. Acad. Sci. U.S.A. 115, E172–E179 (2018). 29279396 10.1073/pnas.1715464115 PMC5777057

[54] X. Cong, Y. Liu, W. Liu, X. Liang, D. H. Russell, A. Laganowsky, Determining membrane protein–lipid binding thermodynamics using native mass spectrometry. J. Am. Chem. Soc. 138, 4346–4349 (2016). 27015007 10.1021/jacs.6b01771

[55] Y. R. Alugubelli, J. Xiao, K. Khatua, S. Kumar, L. Sun, Y. Ma, X. R. Ma, V. R. Vulupala, S. Atla, L. R. Blankenship, D. Coleman, X. Xie, B. W. Neuman, W. R. Liu, S. Xu, Discovery of first-in-class PROTAC degraders of SARS-CoV-2 main protease. J. Med. Chem. 67, 6495–6507 (2024). 38608245 10.1021/acs.jmedchem.3c02416 PMC11056980

[56] R. Dennington, T. A. Keith, J. M. Millam, GaussView 6 (Semichem Inc., 2016).

