# Supplementary material for: From vibrations to function: Spectroscopic detection and quantification of π-π stacking in drug-responsive protein complexes
Source: Sci Adv. 2026 Apr 8;12(15):eaeb3917. doi: 10.1126/sciadv.aeb3917 (PMC13154015; doi:10.1126/sciadv.aeb3917)
Supplement: Supplementary file 1 — Supplementary Text Table S1 Figs. S1 to S13 [file sciadv.aeb3917_sm.pdf]

Science Advances
AAAS

Supplementary Materials for

From vibrations to function: Spectroscopic detection and quantification of π-π stacking in drug-responsive protein complexes

Narangerel Altangerel *et al.*

Corresponding author: Vladislav V. Yakovlev, vladislav.yakovlev.tamu@gmail.com

Sci. Adv. **12**, eaeb3917 (2026)
DOI: 10.1126/sciadv.aeb3917

This PDF file includes:

Supplementary Text
Table S1
Figs. S1 to S13

Supplementary text

Table S1 provides a comparative analysis of existing detection techniques and highlights TRIP's advantages in resolving π-π interactions with high resolution, minimal sample requirements, and direct measurement capabilities *(5, 12-21).*

Techniques	Complex sample	Label free	Physiological relevance	Non destructive	resolution	Sample state
X-ray crystallography	No	Yes*	low	No	Atomic	Crystalline solid
NMR	No	Yes	moderate	Yes	Atomic	Aqueous solution
Cryo-EM	No	Yes	moderate	No	Atomic	Frozen hydrated
Mass spectrometry	No	No	low	no	Protein complex	Gas and solution
Molecular dynamics	No	Yes	moderate	Yes, computational	Atomic (predicted)	Computational model
Fluorescence spectroscopy	limited	No	moderate	yes	Protein complex	Aqueous Solution with fluorescent tag
UV spectroscopy	limited	No	moderate	Yes	Protein complex	Aqueous Solution with special tag
UV Resonance Raman spectroscopy	Yes	Yes	moderate	No	Atomic	Aqueous solution
Surface enhanced Raman	No	Yes	moderate	no	Atomic	Aqueous Solution with nanoparticles
TRIP	Yes	Yes	high	Yes	Atomic	Native solution (PBS)

TRIP requires only 10 μL of sample per measurement, with each sample containing as few as 600 protein molecules, making it the most sensitive method for detecting π-π interactions using spontaneous Raman microscopy. This sensitivity is achieved through an active cooling system that mitigates laser heating, thereby preserving fragile protein samples. Additionally, TRIP does not require complex sample preparation, further streamlining the analysis process.

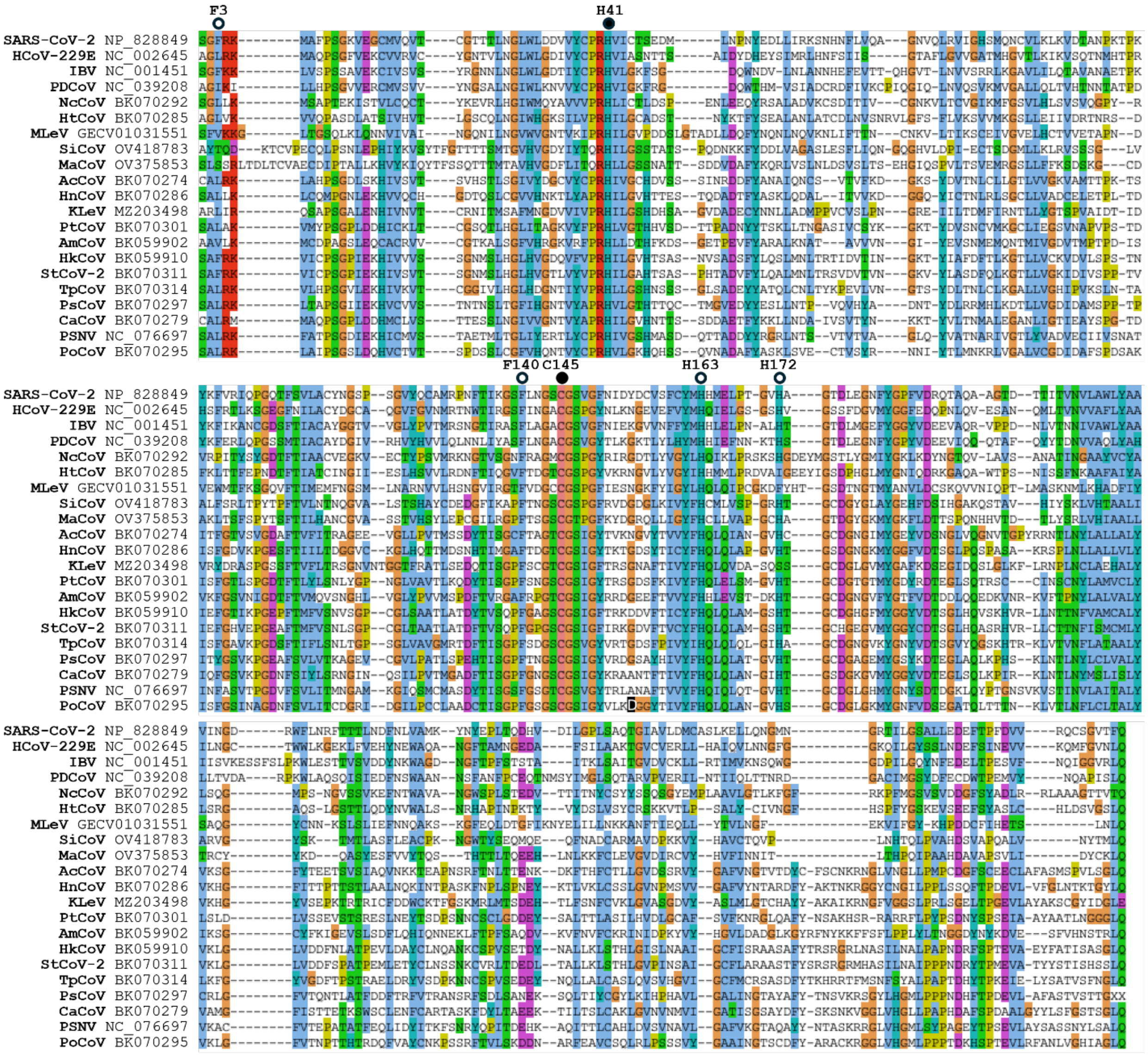

Fig. S1. Conservation of residues involved in pi-pi interactions across the Coronaviridae. Multiple sequence alignment of M^pro from a phylogenetically representative selection coronaviruses, including recently discovered fish and amphibian coronaviruses. Conserved residues of SARS-CoV-2 M^pro are indicated above, with pi-interacting residues marked with white circles and catalytic residues marked with black circles. Coloring is according to both conservation and side-chain properties, using color scheme "Clustal" in Jalview 2.11.4.

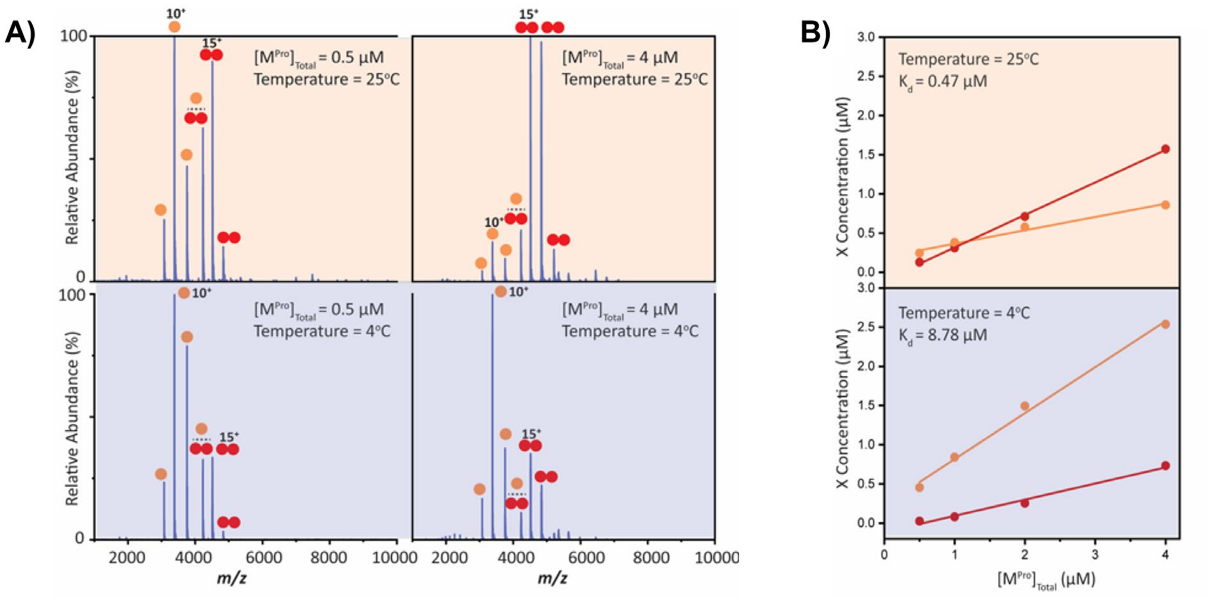

Fig. S2. **Mass spectrometry measurements.** (A) The relative populations of dimers (double red balls) and monomers (orange balls) in the M^{pro} solutions in 4^0C and 25^0C temperatures, (B) M^{pro} concentration dependency graphs for monomer M^{pro} (in orange line) and dimer M^{pro} (in red line) at 4^0C and 25^0C.

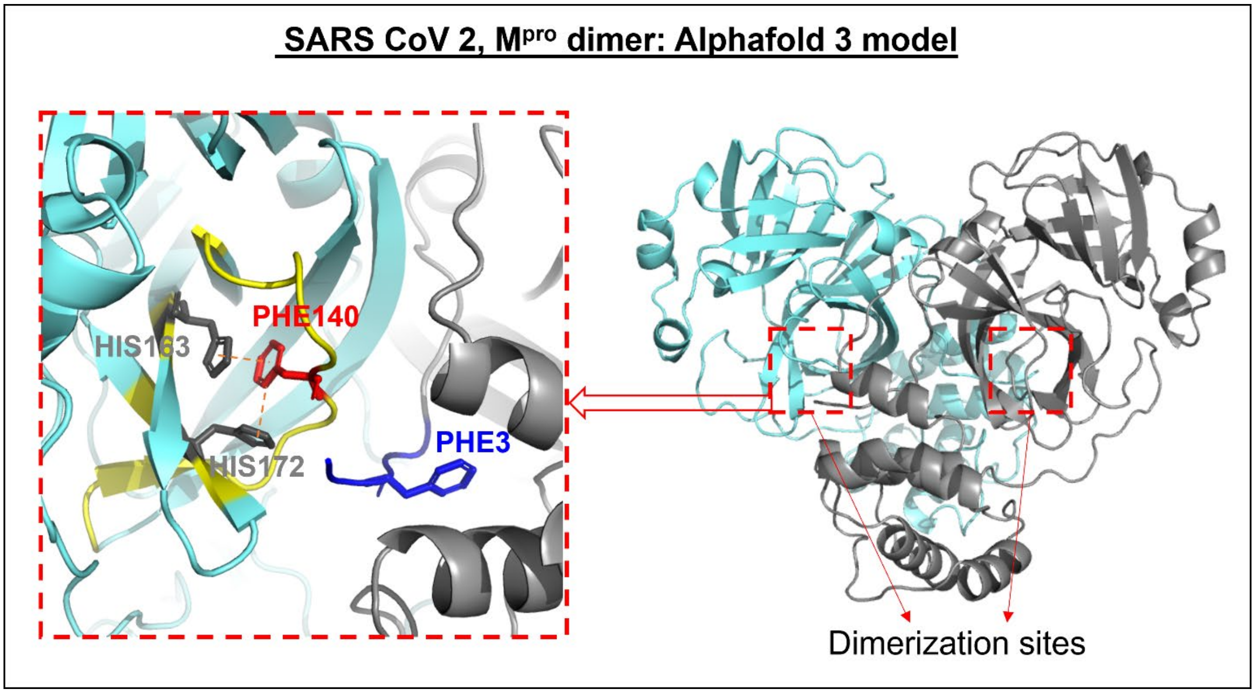

Fig. S3: **Alphafold 3 modeled SARS-CoV 2, M^{pro} ligand-free dimer using its amino acid sequence**. Monomer 1 (Cyan color) and Monomer 2 (grey color); aromatic trimer of PHE140, HIS163 and HIS172.

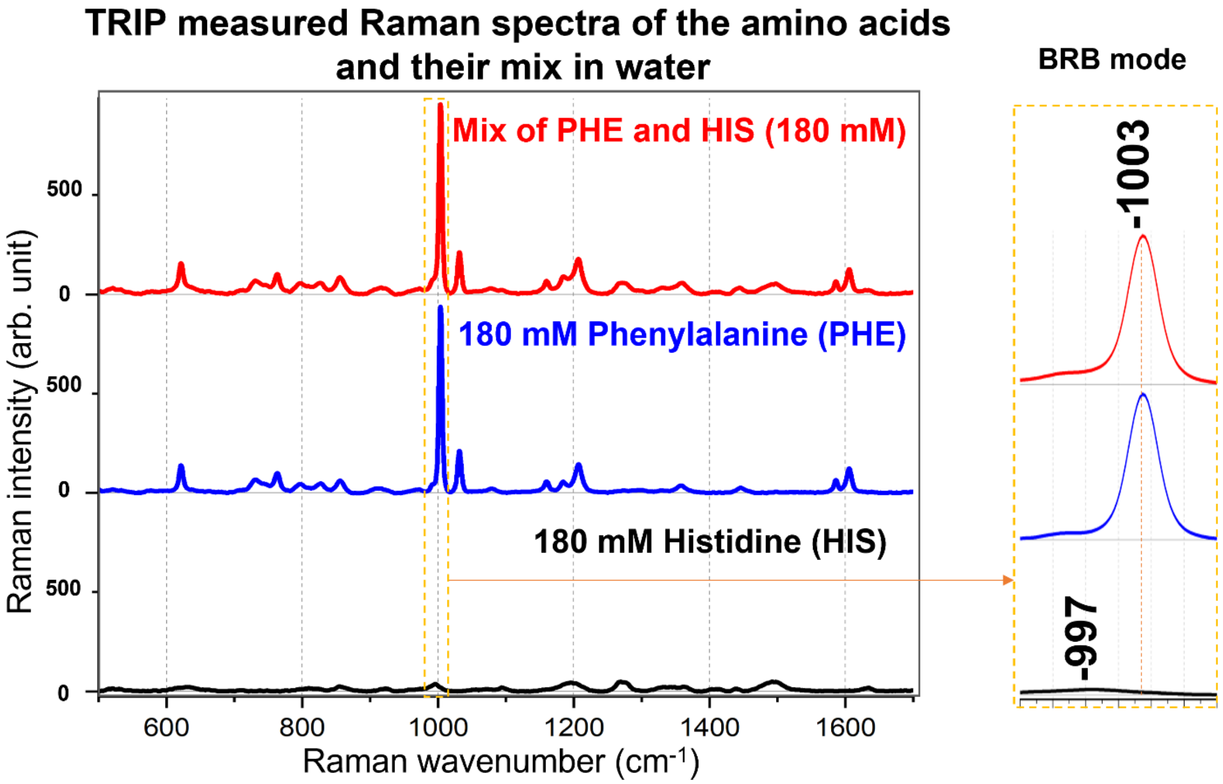

Fig. S4: **TRIP measured Raman spectra.** 180 mM histidine solution (black), 180 mM phenylalanine solution (blue curve), and a 50:50 mix of 180 mM phenylalanine and histidine solution (red curve); zoomed in the benzene ring breathing mode at 1003 cm^{-1}.

Binding (interaction) energy calculations:

For two or more body interactions, the total stabilization energy not simply additive because each ring can influence the electronic distribution of the others.

Two-body interaction energy

For a dimer composed of rings 1 and 2, the total two-body *(41)* binding energy is calculated as:

$$E_{2-\,body} = E_{12} - (E_1 + E_2) \qquad (S1)$$

where

- $E_{12}$is the total energy of the dimer, and
- $E_1$and $E_2$are the energies of the isolated monomers.

A negative value of $E_{2\text{-body}}$indicates a stabilizing interaction between the two rings.

Three-body interaction energy

For a trimer, the true three-body *(41)* cooperative contribution must be isolated from all pairwise interactions. This is obtained using:

$$E_{3-body} = E_{123} - (E_{12} + E_{13} + E_{23}) + (E_1 + E_2 + E_3) \qquad (S2)$$

- E_{123} is the total energy of the trimer,
- E_{12}, E_{13}, and E_{23} are the energies of the three pairwise dimers, and
- E_1, E_2, E_3 are the monomer energies.

A nonzero $E_{\text{3-body}}$ term indicates that the trimer exhibits cooperative effects beyond pairwise additivity—for example, polarization of one ring by the other two, or redistribution of π density across the entire aromatic network

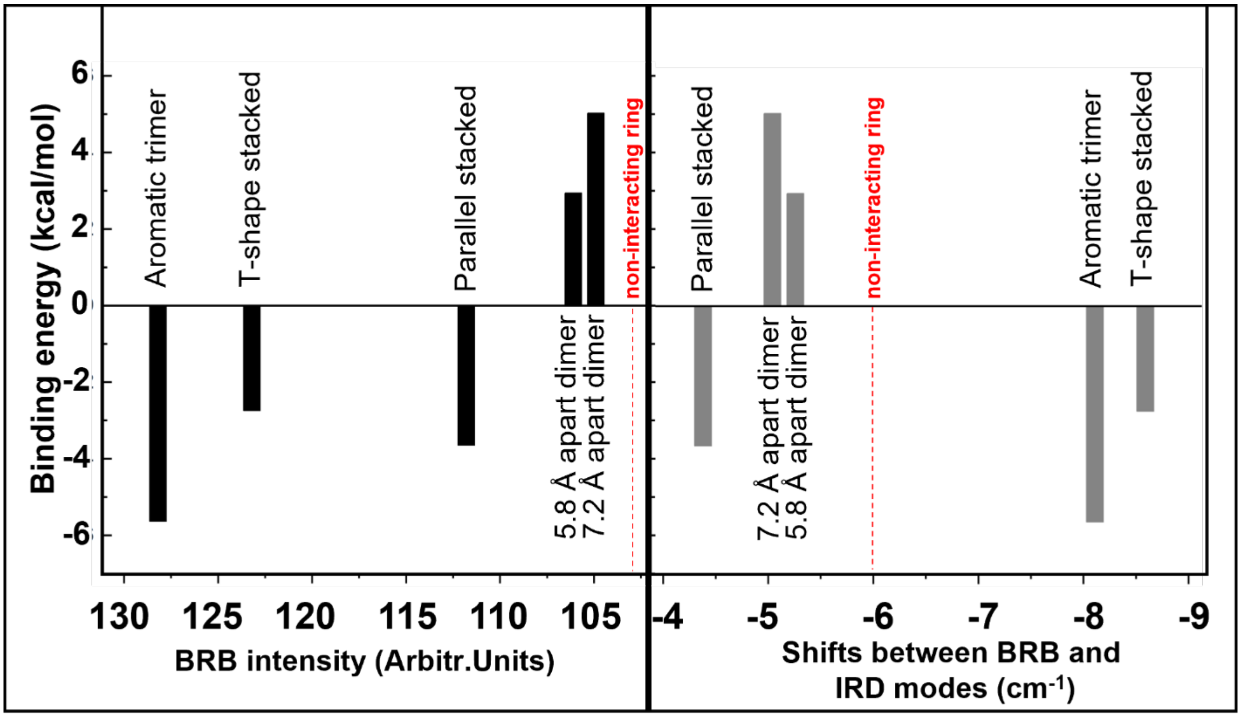

Fig. S5: **Energetic -spectroscopic correlation of π-π stacking**. Binding energy vs BRB intensity and IRD mode shift

Synthesis of VB-B-145:

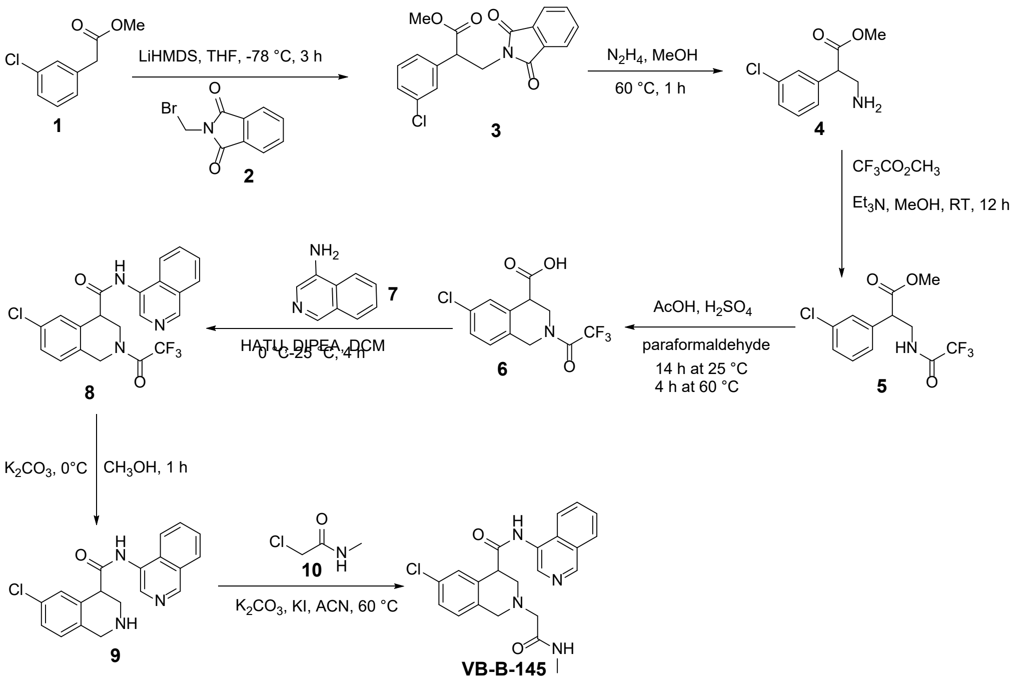

Fig. S6. **Overview of the synthesis of VB-B-145**

Methyl 2-(3-chlorophenyl)-3-(1,3-dioxoisoindolin-2-yl) propanoate (3):

Add a solution of 1 (2.0 g, 10.8 mmol) to a solution of LiHMDS (1M in THF, 13 mL, 1.2 eq.,) at -78 °C in THF (16 mL) over 10 min. Stir the resulting orange mixture for 1 h at -78 °C. Add a solution of N-(bromomethyl) phthalimide (1.2 eq.) in THF (16 mL) dropwise over 10 min. Stir the mixture 1 hour at -78 °C and then at it 1 h at room temperature. Quench the yellow solution with 1N HCl (80 mL). Extract the solution with ethyl acetate. Wash the combined organic layers with water and dry over $MgSO_4$. Concentrate the organic layer. Purify the residue by column chromatography over silica gel (Hex/EA 1:1) to obtain 1.89 g of a white solid.

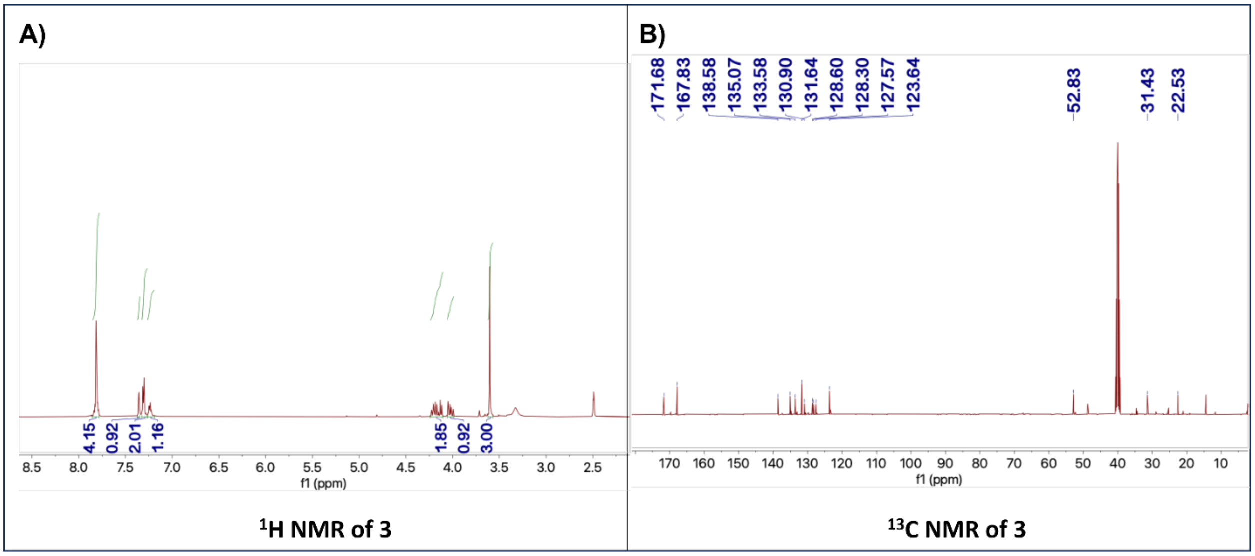

Fig. S7 **NMR data of 3. (A)** ^{1}H NMR (400 MHz, DMSO) δ 7.85 – 7.78 (m, 4H), 7.36-7.34 (m, 1H), 7.33 – 7.29 (m, 2H), 7.27 – 7.21 (m, 1H), 4.24 – 4.10 (m, 2H), 4.02 (dd, *J* = 13.7, 8.5 Hz, 1H), 3.60 (s, 3H). **(B)** ^{13}C NMR (101 MHz, DMSO) δ 171.68, 167.83, 138.58, 135.07, 133.58, 131.64, 130.90, 128.69, 128.30, 127.57, 123.40, 52.83, 31.43, 22.53.

6-chloro-N-(isoquinolin-4-yl)-1,2,3,4-tetrahydroisoquinoline-4-carboxamide (9):

To a suspension of intermediate 3 (1.5, 4.3 mmol) in EtOH (10 mL) was added dropwise hydrazine monohydrate (1.1 mL, 5 eq.) at rt. The mixture was stirred for 2 h at rt. The solvent was removed under reduced pressure and the colorless residue taken up in EA and citric acid 10%. The layers were separated, and the aq. phase was washed with EA. The org. layers were discarded. The product containing aq. phase was basified with NH_4OH and extracted twice with DCM. The combined DCM phases were dried over $MgSO_4$ and concentrated to afford a viscous liquid 4 (650 mg). Add triethylamine (1.5 eq.) and trifluoromethyl acetate (1.05 eq.) to a solution of amine 4 in THF. Stir the reaction at room temperature until completion of the reaction. Concentrate the reaction mixture under reduced pressure to obtain 5 which was used without further purification.

AcOH (0.23 M) and H_2SO_4 (0.35 M) were mixed at 0 °C before 5 (1 eq.) and paraformaldehyde (2 eq.) were added sequentially. The reaction mixture was stirred at room temperature overnight and stir the 4 h at 60 °C, then poured onto H_2O. After extraction with EtOAc (3 x 45 mL), dried over Na_2SO_4, filtered and concentrated *in vacuo*. The crude trifluoroacetate protected 6 tetrahydro isoquinoline was used without further purification.

To this 6 and 7 were dissolved in dry DMF (20 mL) and the reaction was cooled to 0 °C. HATU (1.5 eq.,) and DIPEA (3.0 eq.,) were added, and the reaction mixture was allowed warm up to room temperature and stirred for 12 h. The mixture was then poured into water (50 mL) and extracted with ethyl acetate (4×20 mL). saturated aqueous $NaHCO_3$ (2×20 mL), brine (2×20 mL) and dried over Na_2SO_4. The organic phase was evaporated to dryness and the crude trifluoroacetate protected 8 from the previous step was dissolved in MeOH (0.1 M) and an aqueous K_2CO_3 solution (0.44 M, 3 eq.) was added. The reaction mixture was stirred at 1 h 0°C before being acidified to pH 8 with HCl (1 M). This mixture was extracted with EtOAc and the combined organic layers were washed with H_2O, dried over Na_2SO_4, filtered and concentrated *in vacuo*. the crude material purified by silica gel column chromatography (Hex/EA 2:8) to afford 9 as white solid (120 mg, 54%).

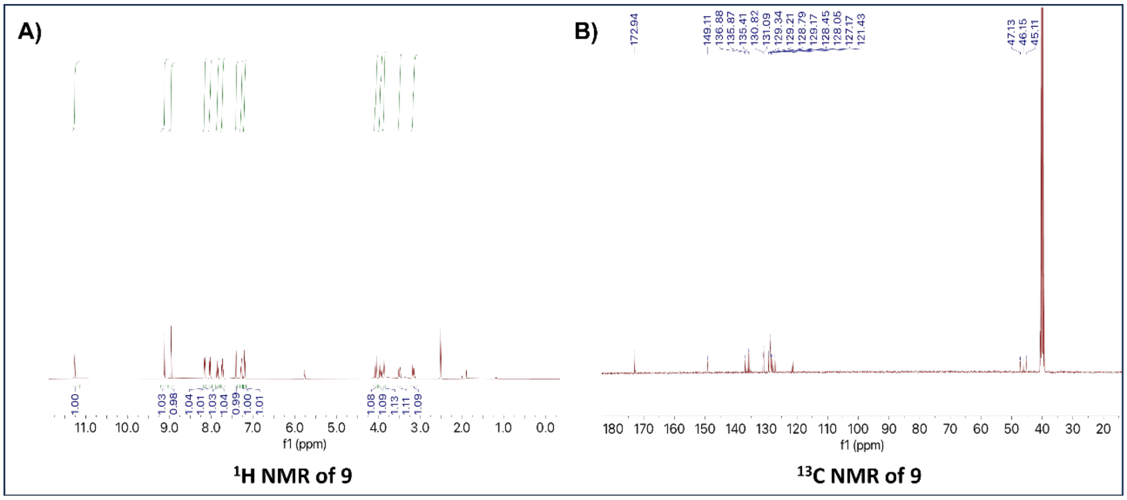

Fig. S8 **NMR data of 9. (A)** ^{1}H NMR (400 MHz, DMSO) δ 11.26 (s, 1H), 9.12 (s, 1H), 8.96 (s, 1H), 8.16 (d, *J* = 8.2 Hz, 1H), 8.03 (dd, *J* = 8.4, 1.2 Hz, 1H), 7.88-7.81 (m, 1H), 7.75-7.70 (m, 1H), 7.40 (d, *J* = 2.2 Hz, 1H), 7.28 (dd, *J* = 8.2, 2.3 Hz, 1H), 7.20 (d, *J* = 8.3 Hz, 1H), 4.07 (d, *J* = 16.3 Hz, 1H), 3.94 (d, *J* = 16.4 Hz, 1H), 3.86 (t, *J* = 4.0 Hz, 1H), 3.49 (dd, *J* = 12.8, 3.5 Hz, 1H), 3.16 (dd, *J* = 12.8, 4.5 Hz, 1H); **(B)** ^{13}C NMR (101 MHz, DMSO) δ 172.94, 149.11, 136.88, 135.87, 135.41, 131.09, 130.82, 129.34, 129.21, 129.17, 128.79, 128.45, 128.05, 127.17, 121.43, 47.13, 46.15, 45.11.

6-chloro-N-(isoquinolin-4-yl)-2-(2-(methylamino)-2-oxoethyl)-1,2,3,4-tetrahydroisoquinoline-4-carboxamide (VB-B-145):

To a solution of 9 (34 mg, 0.1 mmol) in acetonitrile (5 mL) were sequentially added potassium carbonate (14 mg, 0.1 mmol), KI (0.1 eq.,) and 10 (13.5 mg, 0.12 mmol). The reaction mixture was stirred at 60°C for 4 h, then partitioned between EtOAc (20 mL) and water (15 mL). The

organic layer was dried ($MgSO_4$) filtered and concentrated *in vacuo*. the crude material purified by silica gel column chromatography (DCM/MeOH 9:1) to afford VB-B-145 as white solid (25 mg).

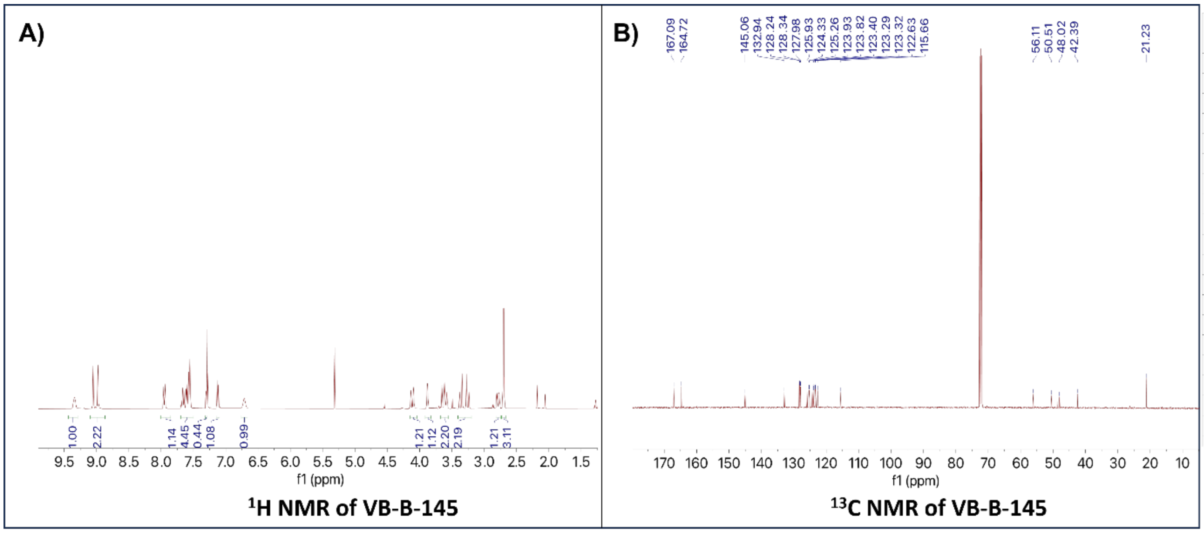

Fig. S9: **NMR data of VB-B-145. (A)** ^{1}H NMR (400 MHz, DMSO) δ 9.35 (d, *J* = 7.7 Hz, 1H), 9.01 (d, *J* = 29.9 Hz, 2H), 7.95 (d, *J* = 8.1 Hz, 1H), 7.70 – 7.47 (m, 4H), 7.12 (d, *J* = 8.4 Hz, 1H), 6.71 (s, 1H), 4.11 (dd, *J* = 15.4, 4.0 Hz, 1H), 3.88 (t, *J* = 2.8 Hz, 1H), 3.76 – 3.53 (m, 2H), 3.34 (s, 1H), 3.26 (d, *J* = 15.7 Hz, 1H), 2.79 (dd, *J* = 11.8, 3.7 Hz, 1H), 2.71 (s, 3H); **(B)** ^{13}C NMR (101 MHz, DMSO) δ 167.09, 164.72, 145.06, 132.94, 128.34, 128.24, 127.98, 125.93, 125.26, 124.33, 123.93, 123.82, 123.40, 123.32, 123.29, 122.63, 115.66, 56.11, 50.51, 48.02, 42.39, 21.23.

<u>VB-B-145 modeling:</u>

VB-B-145 was docked to M^{pro} with Schrodinger Desmond MD simulation program with 10 ns simulation time. The simulated poses of VB-B-145 were evaluated for interactions with catalytic residues including HIS41 and CYS145. Simulated poses of VB-B-145 can be found in the Figure S9 (A-D), where VB-B-145 maintained a strong dual H-bonding with Glu(E)166 via the two amides.

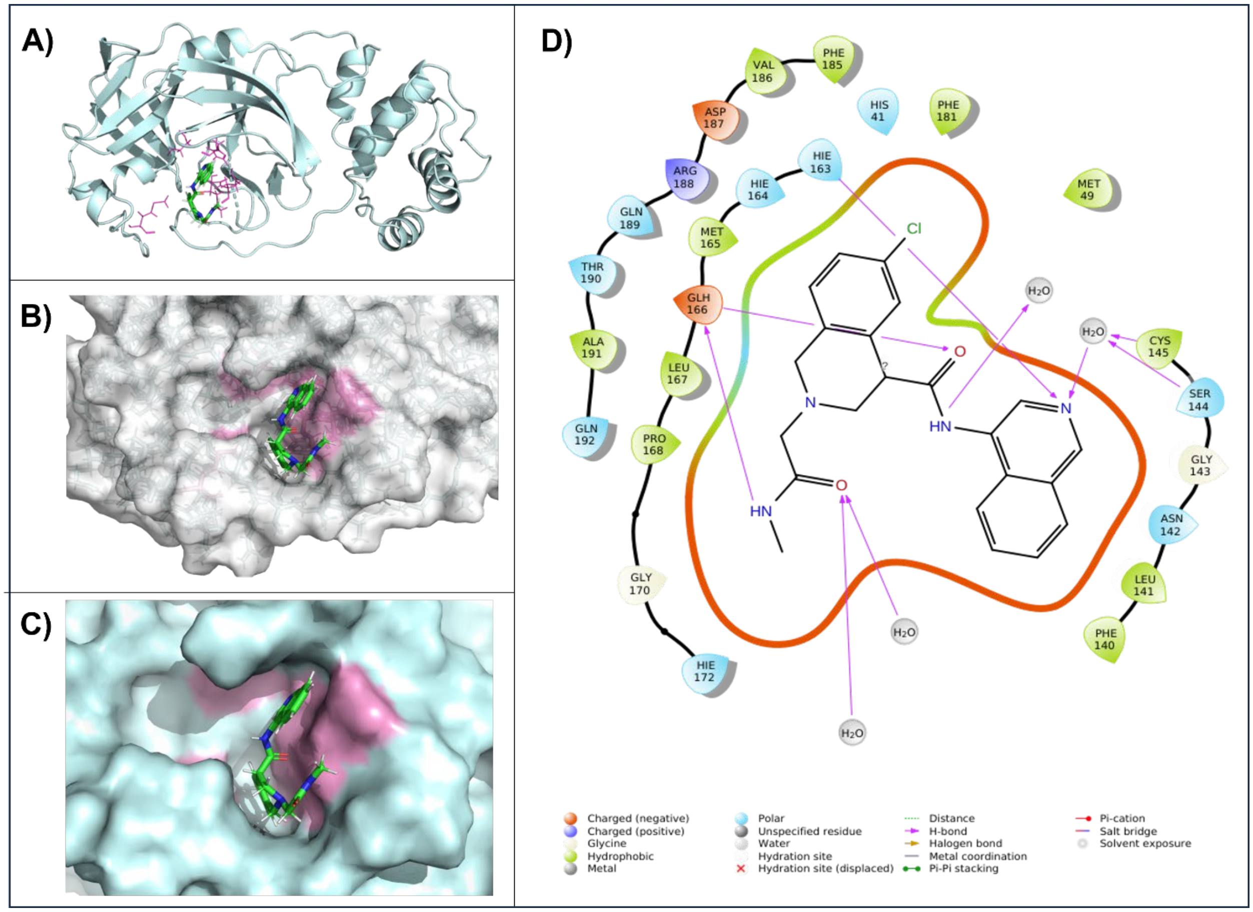

Fig. S10: **The simulated VB-B-145 interactions with monomeric M^{pro}**. **(A)** Cartoon diagram of M^{pro} (pale cyan) showing the VB-B-145 (green sticks) together with MET49, SER144, CYS145, HIS163, GLU166, and GLN189 (magenta sticks) in the active site. **(B)** Solvent-accessible surface of the binding site (white mesh and semi-transparent surface) with the VB-B-145 (green sticks) nestled in a mostly hydrophobic recess (magenta shading). **(C)** Close-up surface view (solid pale cyan) of VB-B-145 (green sticks) in the active site, highlighting shape complementarity between the inhibitor and surrounding residues. **(D)** 2D interaction map (LigPlot-style) of the inhibitor–protein contacts: hydrogen bonds (magenta dashed lines), halide coordination to Cl^- (green line), water-mediated bridges (grey circles), π–π stacking (orange arcs), and hydrophobic contacts (green arcs). Residues are colored by type—charged (orange), polar (cyan), hydrophobic (light green), and aromatic (blue)—and annotated with their sequence positions; distances are given in Å. HIE163 and 164 are neutral histidines that donate ε-nitrogen hydrogen bonds (magenta dashed *lines), GLH166 is a fully protonated glutamic acid that donates a hydrogen bond to VB-B-145 (magenta dashed lines), and HIS41 does not have an explicit tautomer assignment.*

While the α-Nitrogen on the iso-quinoline group forms an additional hydrogen bond with His(H)163 (Figure S9 D), two more residues, Cys(C) 145 and Ser(S)144 forms another hydrogen bond via water bridge that contacts the same Nitrogen (Figure S9) During the simulation, VB-B-145 maintains a < 2Å RMSD from the active site, indicating consistent binding pocket comparing with the co-crystalized ligand.

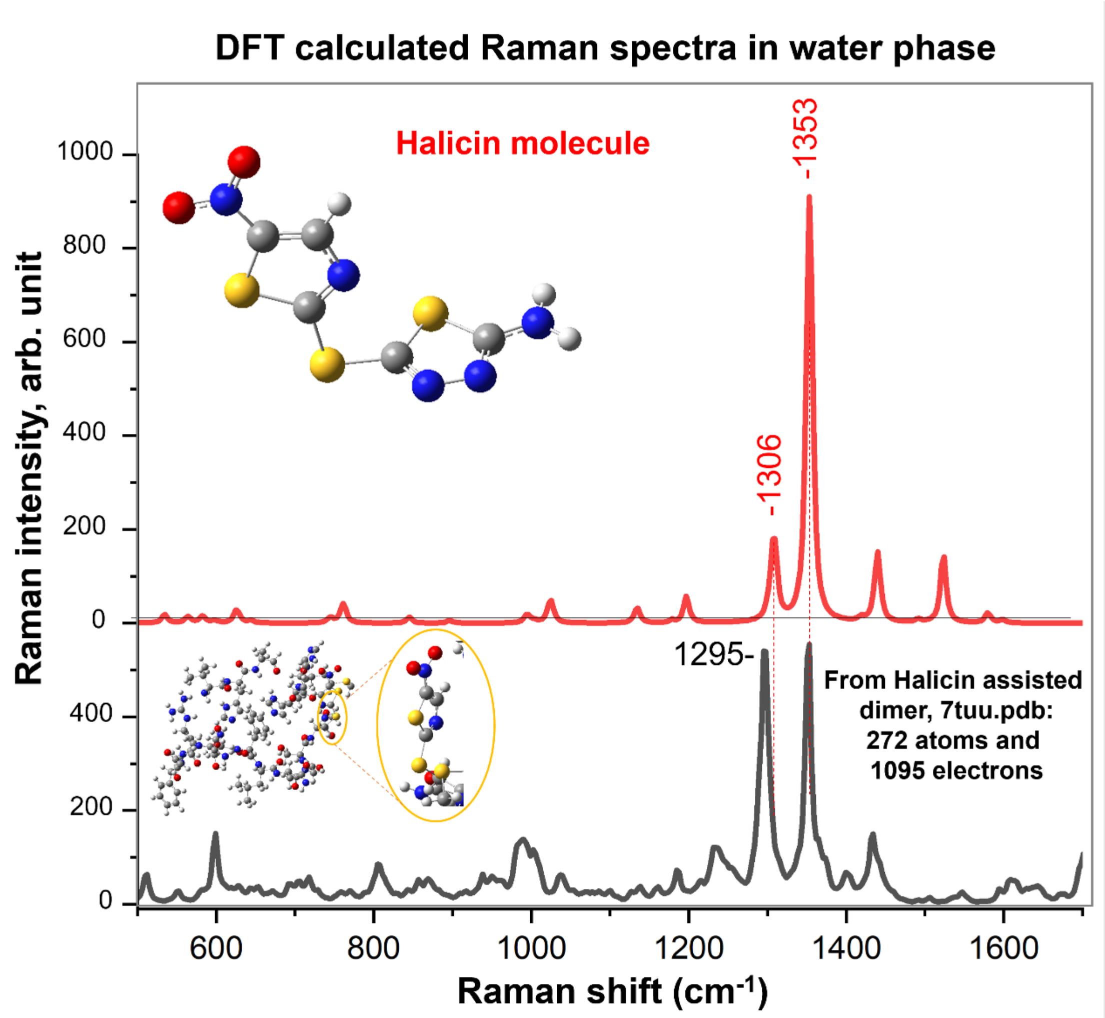

Fig. S11. **DFT (Gaussian 16/B97D/TZVP) calculated Raman spectra**. Free Halicin molecule (red curve) and Halicin assisted dimer, 7tuu.pdb (black curve)

π–π Stacking Strength Influences M^{pro} Composition and Secondary Structure

Amino Acid Profile Stability Correlates with Strong π–π Stacking

To further probe the structural consequences of π–π stacking interactions in Mpro, we applied multiple linear regression (MLR) to TRIP-derived Raman spectra. This technique models the average Raman spectrum of Mpro as a linear combination of reference spectra from individual amino acids and protein standards, enabling the deconvolution of amino acid composition and secondary structure. Previously validated MLR parameters estimated monomeric Mpro's composition with root mean square errors (RMSE) of 1.47% (amino acids) and 3.86% (secondary structure), and were applied here to assess ligand-induced changes (48).

Histogram comparisons (Figure S11A) revealed that dimeric M^{pro} samples—particularly those formed with VB-B-145 and halicin—exhibited compositional changes similar to those of ligand-free dimers, including modest reductions in alanine, glycine, and threonine content.

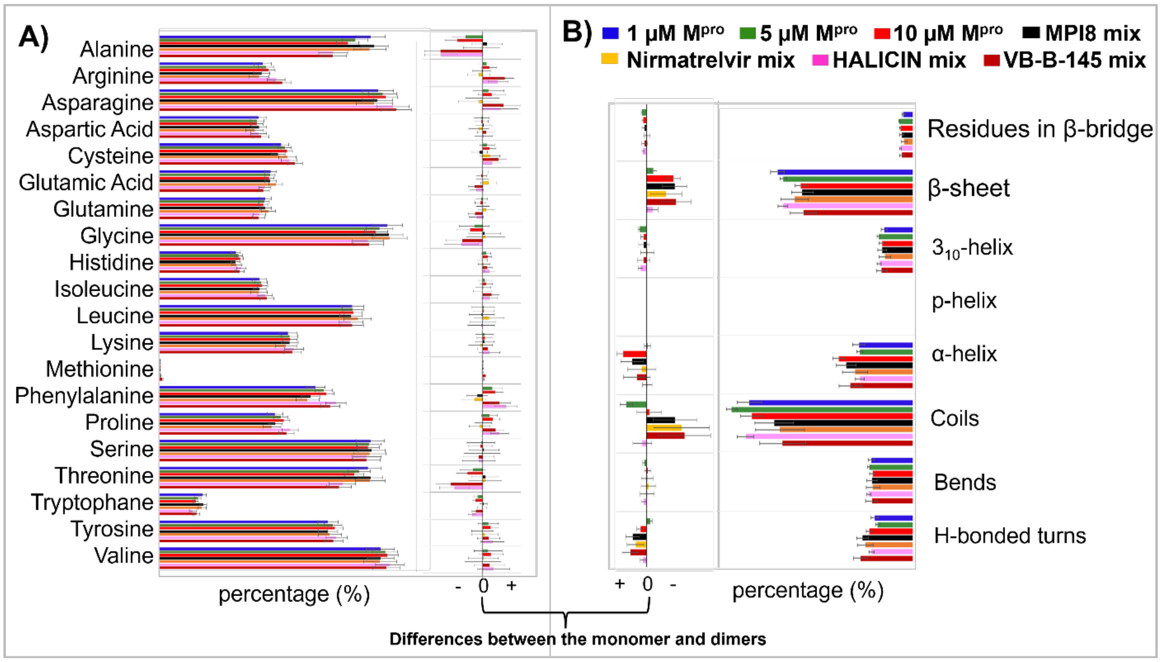

Fig. S12. **SARS-CoV-2 M^{pro} solutions. (A)** Histograms of amino acid frequencies of 1 μM (in blue bars), 5 μM (in green bars), and 10 μM (in red bars), mpi8 mix (in black bars), nirmatrelvir mix (in orange bars), halicin mix (in pink bars), VB-B0145 mix (maroon), and the difference bars between the monomer (1 μM) and each dimers, **(B)** Histograms of secondary structures of 1 μM (in blue bars), 5 μM (in green bars), and 10 μM (in red bars) mpi8 mix (in black bars), nirmatrelvir mix (in orange bars), halicin mix (in pink bars), VB-B0145 mix (in maroon bars), and the difference bars between the monomer (1 μM) and each dimers.

These amino acids, which have weaker Raman signatures, may reflect alterations in surface exposure or conformational accessibility. In contrast, mpi8- and nirmatrelvir-bound M^{pro} samples showed negligible changes in amino acid profiles, likely due to their strong stabilization of the π–π interaction network centered at PHE140. Importantly, the consistent levels of aromatic amino acids across all samples—despite large differences in ligand binding behavior—highlight the structural conservation and functional significance of π–π stacking interactions. Their persistence suggests that aromatic residues, especially PHE140, act as anchoring elements for Mpro dimer integrity.

Secondary Structure Reorganization Reflects Stacking-Mediated Stabilization

The secondary structure estimates derived from MLR (Figure S11B) provided further insight into how π–π interactions influence global conformational states of M^{pro}. First, ligand-free dimers and ligand-assisted dimers (excluding halicin) showed consistent trends: a reduction in β-sheet content accompanied by an increase in α-helices. These structural shifts likely result from rearrangements in the protein's backbone geometry as aromatic π–π stacking stabilizes the dimerization interface and constrains conformational flexibility. These observations align with previous CD and SAXS data on M^{pro} folding states and dimerization *(49).*

Second, a notable shift was observed in the balance between hydrogen-bonded turns and random coils. All ligand-bound dimers, with the exception of Halicin, displayed a marked increase in H-bonded turns alongside a pronounced reduction in coil content. This inverse

trend—previously unreported for M^{pro} —indicates that π–π stacking and ligand engagement promote local folding stabilization. The rise in hydrogen-bonded turns likely constrains loop flexibility, reducing disordered regions and enhancing overall structural order. These findings highlight how conserved aromatic interactions contribute not only to interface stabilization but also to broader secondary structure reorganization.

In contrast, halicin-bound dimers showed minimal deviation from the monomeric secondary structure profile, consistent with its weaker disruption of π–π interactions at the dimer interface. The VB-B-145 dimer showed modest but reproducible shifts, also reflecting partial engagement of the aromatic core.

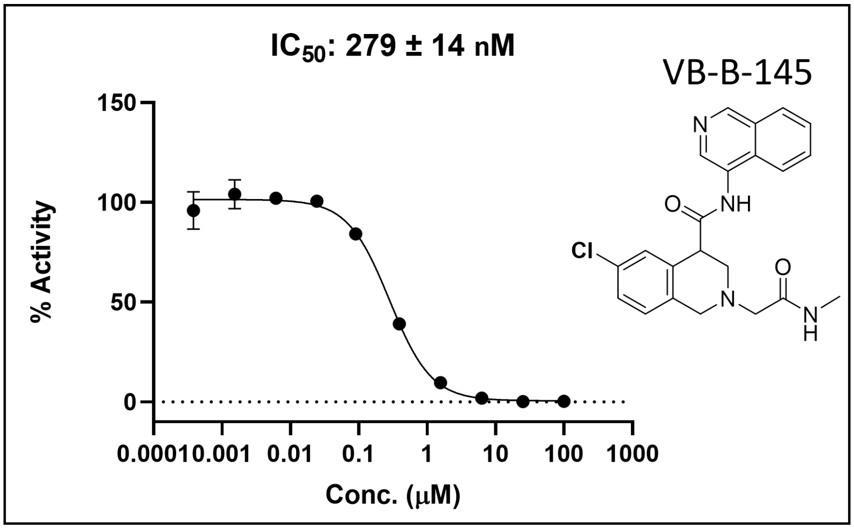

Fig. S13: **Chemical structure and inhibition of M^{pro} activity by VB-B-145**.
